# Dynamic expression of *Ralstonia solanacearum* virulence factors and metabolism-controlling genes during plant infection

**DOI:** 10.1186/s12864-021-07457-w

**Published:** 2021-03-09

**Authors:** R. de Pedro-Jové, M. Puigvert, P. Sebastià, A. P. Macho, J. S. Monteiro, N. S. Coll, J. C. Setúbal, M. Valls

**Affiliations:** 1 Department of Genetics, University of Barcelona, Barcelona, Catalonia Spain; 2 Centre for Research in Agricultural Genomics (CSIC-IRTA-UAB-UB), Bellaterra, Catalonia Spain; 3 Shanghai Center for Plant Stress Biology, CAS Center for Excellence in Molecular Plant Sciences, Chinese Academy of Sciences, Shanghai, 201602 China; 4 Departamento de Bioquímica, Universidade de São Paulo, São Paulo, Brazil

**Keywords:** *Ralstonia solanacearum*, Bacterial wilt, RNAseq, Virulence factors, Dynamic gene expression, Metabolism, T3SS, Effectors, Xylem, Apoplast

## Abstract

**Background:**

*Ralstonia solanacearum* is the causal agent of bacterial wilt, a devastating plant disease responsible for serious economic losses especially on potato, tomato, and other solanaceous plant species in temperate countries. In *R. solanacearum*, gene expression analysis has been key to unravel many virulence determinants as well as their regulatory networks. However, most of these assays have been performed using either bacteria grown in minimal medium or *in planta*, after symptom onset, which occurs at late stages of colonization*.* Thus, little is known about the genetic program that coordinates virulence gene expression and metabolic adaptation along the different stages of plant infection by *R. solanacearum*.

**Results:**

We performed an RNA-sequencing analysis of the transcriptome of bacteria recovered from potato apoplast and from the xylem of asymptomatic or wilted potato plants, which correspond to three different conditions (Apoplast, Early and Late xylem). Our results show dynamic expression of metabolism-controlling genes and virulence factors during parasitic growth inside the plant. Flagellar motility genes were especially up-regulated in the apoplast and twitching motility genes showed a more sustained expression *in planta* regardless of the condition. Xylem-induced genes included virulence genes, such as the type III secretion system (T3SS) and most of its related effectors and nitrogen utilisation genes. The upstream regulators of the T3SS were exclusively up-regulated in the apoplast, preceding the induction of their downstream targets. Finally, a large subset of genes involved in central metabolism was exclusively down-regulated in the xylem at late infection stages.

**Conclusions:**

This is the first report describing *R. solanacearum* dynamic transcriptional changes within the plant during infection. Our data define four main genetic programmes that define gene pathogen physiology during plant colonisation. The described expression of virulence genes, which might reflect bacterial states in different infection stages, provides key information on the *R. solanacearum* potato infection process.

**Supplementary Information:**

The online version contains supplementary material available at 10.1186/s12864-021-07457-w.

## Background

Brown rot or bacterial wilt of potato is a vascular disease caused by the bacterial phytopathogen *Ralstonia solanacearum*. This gram-negative β-proteobacterium is among the most threatening bacterial phytopathogens worldwide, as it can infect over 200 different plant species, including many important crops such as potato, tomato, peanut, eggplant and banana [1–3]. Although *R. solanacearum* is endemic of tropical and sub-tropical regions, phylotype IIB-1 strains such as UY031 are acclimated to lower temperatures and have caused important outbreaks in temperate areas [4–6].

*R. solanacearum* has a complex life cycle. The pathogen survives in soil and water for long periods of time [7]. When *R. solanacearum* senses the roots of natural hosts by plant exudates [8], it penetrates the host through the root elongation zone, root wounds or secondary root emerging points [9]. The root intercellular spaces (the apoplast) constitutes a front line in the arms race in plant-pathogen interactions and it is thus a hostile environment to phytopathogens [10]. Therefore, colonisation of the apoplast is key for *R. solanacearum* pathogenicity [11–13]. Successful infections involve entry into the vascular cylinder and extensive colonisation of the xylem vessels [14, 15]. Occlusion of the vasculature due to massive exopolysaccharide (EPS) production and bacterial multiplication ultimately cause wilting symptoms and plant death [9, 16].

To progress across the different plant tissues, *R. solanacearum* uses a panoply of virulence determinants [17, 18]. The main virulence factor in this and many other pathogenic bacteria is the Type III Secretion System (T3SS) [19, 20], which delivers effector proteins inside the plant cells, hijacking the cellular machinery for bacterial benefit [21]. Another key virulence determinant is EPS. EPS leads to the clogging of the xylem vessels and plant symptom appearance, and it can also bind to the cell wall and protect the bacterium from plant defences [22, 23]. In addition, the general secretion system (type II) secretes important virulence factors into the apoplast, including cell wall degrading enzymes [24]. These enzymes are collectively important for *R. solanacearum* plant colonisation, since multiple deletion of the *egl*, *pehA/B/C, pme* and *cbhA* genes compromised pathogenicity [25]. Bacterial motility also plays important roles during parasitic life *in planta*. For instance, *R. solanacearum* flagellar components were shown to be essential at early stages of infection [26] and mutants in the main twitching gene *pilA* were less pathogenic [27]. On the other hand, the *R. solanacearum* genome encodes the necessary enzymes to use nitrate as an energy source (i.e. dissimilatory nitrate reduction), to incorporate nitrate as a molecular building block (i.e. assimilatory nitrate reduction) [28] and to detoxify reactive nitrogen species (i.e. denitrification) [29]. The ability to use nitrate as terminal electron acceptor has been proposed to sustain rapid bacterial growth in the xylem, a hypoxic environment that is nonetheless rich in nitrate [29, 30].

Gene regulation analyses are essential to decipher how *R. solanacearum* finely tunes its pathogenicity. For instance, transcription of the *hrp* genes -encoding the T3SS- and its related effectors was found to be controlled by the HrpB transcriptional activator. HrpB lies downstream of a regulatory cascade induced by bacterial contact with the plant cell wall [31] . The cascade includes the membrane receptor PrhA, the signal transducer PrhI and the transcriptional regulators PrhJ and HrpG, the latter directly activating *hrpB* transcription [32, 33]. Gene expression studies demonstrated that the *R. solanacearum hrp* genes and T3SS effectors were transcribed *in planta* at late infection stages [34]. Based on these results, it was speculated that *R. solanacearum* could inject T3Es to the xylem parenchyma cells in order to hijack plant defences and manipulate the host metabolism [34]. These findings were later confirmed in gene expression studies using bacteria extracted from infected tomato and banana plants [14, 35] or bacterial transcripts isolated from infected potato roots [36]. Similar to the T3SS, EPS production is also stringently controlled through the expression of the *eps* operon, which encodes all EPS biosynthesis genes [37]. The *eps* operon promoter is dependent on the global regulator PhcA, whose production is induced at bacterial densities above 10^7^ CFU/ml [37–39] . Finally, it has been described that some crosstalk exists between the *eps* and the *hrp* gene regulation, since *hrpG* is negatively regulated by *phcA* [32, 40].

Bacterial interactions in plant hosts do not consist on one static phase, but rather in a dynamic interaction during disease development. However, all *R. solanacearum in planta* transcriptomic studies have focused so far on a specific stage of the infection process: xylem colonisation at the onset of disease symptoms [14, 35, 41, 42], with the exception of a single study indirectly analysing bacterial reads from infected roots [36]. Among the differentially expressed (DE) genes identified in these previous studies, the T3SS, T3Es, motility genes, ROS detoxifying enzymes and cell wall degrading enzymes were found up-regulated in most cases. Dynamic transcriptomic studies of the model plant pathogen *Pseudomonas syringae* analysing different moments of the disease development have recently revealed a changing bacterial behaviour. For example, flagellar motility and chemotaxis-related genes were transcribed in the epiphytic phase, while genes controlling metabolism were expressed in the apoplast [43]. In another study, gene expression of virulent and avirulent *P. syringae* strains was studied at different time points after inoculation of various *Arabidopsis thaliana* defence-related mutants. This work identified an iron response regulator that was induced at early infection stages, counteracting plant immunity [44]. Other time course transcriptomes in *P. syringae* have described an up-regulation of flagellar, chemotaxis and two-component system genes and a down-regulation of bacterial secretion systems and general metabolism at late infection stages in bacteria recovered from plants with pre-induced immunity compared to naïve plants [45]. Together, these studies have started revealing the complex landscape of transcriptomic changes occurring over time during the course of a bacterial infection.

Due to the various environments it encounters along the infection process and because of its economic relevance, *R. solanacearum* is an excellent model to analyse gene expression in different plant tissues, which correspond to distinct phases of the infection process. Here, we have analysed the transcriptome of the cold adapted *R. solanacearum* UY031 at three different conditions. We have used the economically important crop potato plant where the *R. solanacearum* UY031 was naturally identified a decade ago [46]. Our data clearly shows that *R. solanacearum* genes behave dynamically inside the plant during the course of infection. We have identified condition-specific expression of virulence and metabolic genes, providing a new dynamic perspective of the *R. solanacearum* infection process.

## Results

### *R. solanacearum* transcriptomes reflect four main genetic programmes inside the plant

**Fig. 1:**
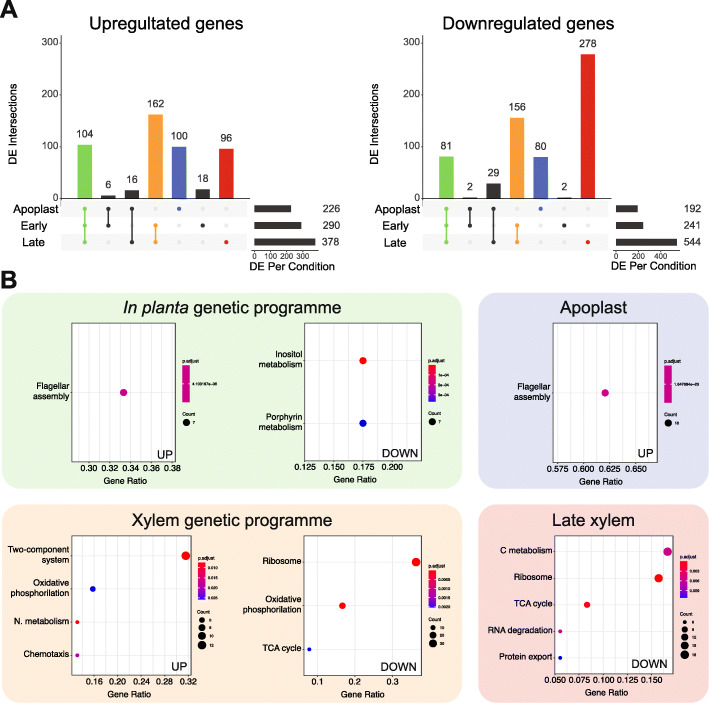
**Transcriptomic profile of** ***R. solanacearum*** **UY031** ***in planta***. **a**. Shared and unique DE genes across the three *in planta* conditions for the up-regulated (left) and down-regulated (right) genes. Each vertical bar plot represents the number of shared DE between the conditions indicated by the lines and dots in the schematic below. The horizontal bar plots on the right indicate the total DE genes per *in planta* condition compared to rich medium. **b.** For the intersection of Apoplast, Early and Late (*in planta* genetic programme), Early and Late (Xylem genetic programme), Apoplast and Late xylem alone, the list of genes was extracted and surveyed for enriched KEGG pathways. Dot plots of the enriched KEGG pathways for the up- (left) and down-regulated (right) genes in each environment are shown below. DE genes were identified with DEseq2 (*p-adj* > 0.01, log_2_ FC ± 1.5) and plotted using the R package UpsetR

To elucidate the genes deployed by *R. solanacearum* throughout infection, we profiled the gene expression of strain UY031 in its natural susceptible potato host. We collected bacterial samples from the apoplast —a condition mimicking early root infection, when the bacterium traverses and multiplies in this compartment [47]— and from the xylem of infected plants at six and ten days post-inoculation, which correspond to the onset of the disease (early xylem) or to the final stages when plants are completely wilted (late xylem) (Additional File 1 B and 2A). *R. solanacearum* plant infection through roots is highly variable due to stochastic changes in the physiological state of the plant, the initial inoculum and available root entry sites. To overcome this problem, we took advantage of a luminescent *R. solanacearum* reporter strain previously developed in our group to measure bacterial colonisation and we normalized values for tissues containing comparable bacterial loads at different times of infection [48]. The *in planta* transcriptomes were compared with that obtained from bacteria grown in liquid rich B medium, a reference condition known to repress many of the pathogen’s virulence determinants [49]. Principal component analysis (PCA) of the transcripts from each sample showed a clear clustering of the biological replicates and a clear differentiation of the xylem samples from the reference and apoplast samples (PC1, explaining 65% of the variation) (Additional File 2 C). Comparison of the *in planta* transcriptomes with that obtained in axenic growth in rich medium identified 418 differentially expressed genes (DEGs) in the apoplast, 531 in the early xylem and 922 in the late xylem (log_2_ fold change ≥ |1.5| and adjusted *p-value* ≤ 0.01). Of these genes, 226 and 192 were up- and down-regulated, respectively, in the apoplast, 290 and 241 in the early xylem, and 378 and 544 in the late xylem (Fig. 1a and Additional File 3).

Comparison of the DEGs in each *in planta* condition is in agreement with the previously published *R. solanacearum in planta* transcriptomic studies (Additional File 4 A). DE transcripts from the same UY031 strain retrieved from total RNAs of infected wild potato roots [36] showed up to 17–18% overlap with the apoplast condition and lower overlap with the other conditions assayed in the present study, and the gene expression values showed a high correlation (Additional File 4 B). This is logical, since the transcriptome previously obtained from roots of asymptomatic plants corresponds to an early time of infection where most bacteria grow apoplastically and only a small proportion of bacteria have already reached the xylem. The highest overlap (34% overlap in up- and 36% in down-regulated genes, respectively) was found between our early xylem conditions and the microarray transcriptome of the phylogenetically close strain UW551 isolated from tomato plants at a comparable infection time (onset of wilting symptoms) [14], which further validates our results (Additional File 4). The overlap is obviously lower with comparable transcriptomes obtained using the distantly related GMI1000 strain.

To discover the DEGs common or unique to the different plant environments, we analysed the shared genes among the different conditions studied. As can be observed in Fig. 1a, two intersections (i.e. *in planta* and xylem) and two conditions (i.e. apoplast and late xylem) that correspond to bacterial growth in precise environments included most of the DEGs. On this basis, we defined four genetic programmes where *R. solanacearum* expresses exclusive gene sets: *in planta* (genes shared in all *in planta* conditions: apoplast, early and late xylem), the xylem (genes shared in early and late xylem), the apoplast, and the late xylem. Similarly, DE in all *in planta* conditions were 104 up- and 81 down-regulated genes. The differentially expressed genes in the xylem genetic programme (both time points analysed) included a total of 162 and 156 up- and down-regulated genes. Finally, 100 and 80 genes were, respectively, up- or down-regulated solely in the plant apoplast and 96 and 278 only in the late xylem condition, when plants are mostly dead. The remaining conditions or overlaps between conditions included fewer than 30 specifically DEGs (Fig. 1a) and we did not consider them a proper “genetic programme”. Overall, as hinted by the PCA analysis, the apoplast showed the most divergent transcriptome of the *in planta* conditions, whereas the samples extracted from the xylem (early and late) were the most similar. However, a substantial fraction of genes was only differentially expressed in the late xylem (40% of those DE in this condition).

### *R. solanacearum* upregulates a variety of virulence factors *in planta*

Functional enrichment of gene annotations is a powerful tool to evaluate the genes involved in similar roles or pathways in each experimental condition. Thus, we investigated the enrichment of KEGG pathways and GO terms in the genes that appeared DE in all *in planta* conditions. Since the KEGG database contains metabolic pathways and terms specifically for prokaryotes, we ocused on its categories for enrichment analysis. Among the genes up-regulated in all *in planta* conditions, only the KEGG flagellar assembly pathway was enriched (Fig. 1b). This result was confirmed by the GO enrichment analysis, where the bacterial flagellum-dependent cell motility term was similarly over-represented, together with transposase activity and DNA-mediated transposition (Additional File 5). On the other hand, the enriched KEGG terms amongst the genes down-regulated in all *in planta* conditions were all related to metabolism: inositol phosphate metabolism, and porphyrin and chlorophyll metabolism (Fig. 1b), and the GO term cobalamin biosynthetic process (Additional File 5).

Manual curation of gene annotations enabled us to pinpoint a high number of pathogenicity-related functions up-regulated in all *in planta* conditions. These genes had been overlooked by the global enrichment analysis because virulence genes are not in a KEGG pathway and pathogenicity-related terms in GO are too general and have not been widely used. Thus, we used genomic and bibliographic information to create the gene category “virulence and parasitic fitness” for the UY031 strain and calculated its enrichment in all conditions or genetic programmes analysed in this work (see Methods). The new category included all genes encoding the type III secretion system (T3SS) and its associated effectors (T3Es), genes involved in motility, EPS and phytohormone biosynthesis, ROS scavenging, cell-wall degrading enzymes, and nitrogen metabolism (Additional File 5). As expected, the created “virulence and parasitic fitness” category was clearly enriched in the up-regulated genes in the *in planta* genetic programme (*p-value* = 1.4·10^− 14^). Detailed analysis of the subcategories included in “virulence and parasitic fitness” indicated that T3SS and T3Es (*p-value* = 2.4·10^− 12^) and motility (*p-value* = 5.7·10^− 5^) were also significantly enriched among the up-regulated genes. For instance, 20% (12 out of 60) of the genes annotated as T3Es were overexpressed in all *in planta* conditions. The enriched motility subcategory included a total of 11 genes, containing both flagellar and type IV pili. Similarly, the polygalacturonase gene *pglA,* encoding one out of the six cell-wall degrading enzymes in the genome was also up-regulated in the plant. Other virulence genes up-regulated in bacteria growing in any of the studied *in planta* conditions included *efe,* responsible for ethylene formation, the reactive oxygen species (ROS) scavenging superoxide dismutase *sodC*, and *epsR,* encoding the exopolysaccharide (EPS) repressor. Finally, only the EPS subcategory was under-represented *in planta* (*p-value* = 1.25·10^− 2^), which can be explained by the high expression of the exopolysaccharide synthesis operon in the reference rich medium [38].

### Flagellar genes and the upstream regulators of the T3SS are exclusively up-regulated in the apoplast

Once *R. solanacearum* has infected the roots of a susceptible host plant it must cross the root cortex through the apoplast. The KEGG flagellar assembly pathway was enriched in the genes exclusively up-regulated in the apoplast (Fig. 1b). Similarly, the four GO terms referring to the flagellum (bacterial-type flagellum-dependent cell motility, bacterial-type flagellum basal body, bacterial-type flagellum and bacterial-type flagellum assembly) and phosphopantetheine binding were also enriched in this genetic programme (Additional File 5). A closer perusal of the list of up-regulated genes in the apoplast genetic programme also revealed that the “virulence and parasitic fitness” category was enriched (*p-value* = 4.2·10^− 15^). *PrhJ* and *hrpG*, key upstream regulators of the T3SS activation cascade [31], were up-regulated in this genetic programme. On the other hand, none of the downstream T3SS transcriptional activators and only two of 60 T3E genes (*ripE2* and *ripAD)* were exclusively up-regulated in this genetic programme. None of the KEGG pathways nor GO terms were enriched amongst the genes down-regulated in the apoplast.

### *R. solanacearum* adapts to the xylem environment by inducing virulence, chemotaxis and nitrogen metabolism genes

After travelling through the root apoplast, *R. solanacearum* crosses the Casparian strip, reaching the plant vasculature and heavily colonising the xylem vessels. As mentioned before, a substantial number of *R. solanacearum* genes was DE in the xylem genetic programme, both at early and late conditions (Fig. 1a). Almost one third (12 out of 38) of the genes with associated KEGG pathways differentially up-regulated in the xylem irrespective of the condition belonged to the enriched category two-component system (Fig. 1b). This includes genes that participate in chemotaxis signal transduction, nitrate reduction, and oxidative phosphorylation. Three other categories were enriched in the genes up-regulated in the xylem: oxidative phosphorylation (six genes), bacterial chemotaxis (five genes) and nitrogen metabolism (five genes). The up-regulated nitrogen metabolism genes included nitrate transporters (*nark1*/2), enzymes involved in the denitrification pathway (*aniA*, *norB*) and in the dissimilatory nitrate reduction pathway (*narG/H/I, nirB/D*) as well as in reactive nitrogen species detoxification (*hmpX*). The enriched term bacterial chemotaxis included genes involved in different steps of swimming motility, including membrane chemosensors, signal transduction components (i.e. *cheZ1, cheA, cheR*) and flagellar motor genes (i.e. *motB*). The “virulence and parasitic fitness” category was also enriched in the xylem genetic programme up-regulated genes (*p-value* = 8.8·10^− 5^). Amongst these genes were 9 out of 60 T3Es annotated in strain UY031 genome (*ripAE, ripY, ripAN, ripC1, ripN, ripAP, ripF2, ripBH,* and *ripS5),* and one out of six cell wall degrading enzymes (*pme)*. Other overexpressed genes in the category included 10 motility genes and the cytokinin biosynthesis gene *tzs*. Finally, amongst the 102 KEGG tagged down-regulated genes in the xylem, the enriched categories were: ribosome, oxidative phosphorylation and citrate (TCA) cycle (Fig. 1b). GO enrichment in down-regulated genes similarly showed the over-represented categories translation, ribosome, structural constituent of ribosome, RNA binding, rRNA binding (Additional File 5). In summary, a large set of *R. solanacearum* genes was found DE in the xylem throughout infection, including up-regulation of nitrogen utilisation and virulence genes, such as T3Es and down-regulation of genes encoding the citrate cycle enzymes and the electron transport chain.

### *R. solanacearum* inhibits a large number of metabolic pathways at late infection stages

Besides the DE genes in the xylem throughout infection, a large set of *R. solanacearum* genes was exclusively DE in the Late xylem genetic programme, at late stages of infection when plants are already wilted (Fig. 1a). Surprisingly, no KEGG category was enriched in this abundant set of up-regulated genes, but our “virulence and parasitic fitness” category was enriched in the up-regulated genes (*p-value* = 5·10^− 3^). Within this category, two subcategories were also enriched: T3SS & T3Es, including six effectors, three of the GALA family (*ripG3*, *ripG4* and *ripG6*) (*p-value* = 8.5·10^− 3^), and motility, with six involved in chemosensing and signal transduction (*p-value* = 3.68·10^− 2^). In the genes differentially down-regulated in the late xylem condition, five KEGG categories were enriched: carbon metabolism (18 out of 108 genes tagged), ribosome (17 genes), TCA cycle (9 genes), RNA degradation (six genes) and protein export (six genes) (Fig. 1b). GO enrichment analysis also showed similar results with the overrepresented categories translation, ribosome, structural constituent of ribosome, RNA binding and tricarboxylic acid cycle (Additional File 5). In sum, *R. solanacearum* exclusively downregulates at late infection stages in the xylem a large subset of genes involved in the central metabolism and its derived metabolic pathways.

### Expression profiles reinforce the existence of specific genetic programmes in the apoplast and the xylem

**Fig. 2:**
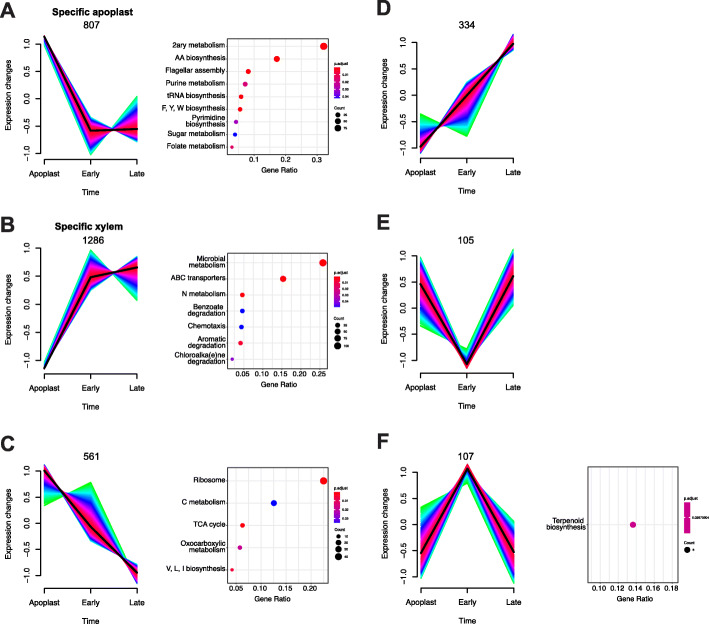
**Gene expression dynamics of** ***R. solanacearum*** **throughout infection**. Six clusters were obtained through Mfuzz clustering of log_2_-fold-change data of the apoplast, early and late xylem conditions normalised to the reference rich liquid media. Clusters include the genes with a membership higher than 70% and consistently associated to the same cluster on at least 30 out of 40 iterations. Number of genes indicated above each graph. The list of genes associated to each cluster was extracted and surveyed for enriched KEGG pathways. Dot plots of the enriched KEGG pathways in each cluster are shown next to the cluster

The findings described so far strongly suggest that *R. solanacearum* expresses specific sets of genes at each step of the infection process. To better understand this dynamic process, we obtained the expression profiles of the *R. solanacearum* UY031 genes in the three *in planta* conditions: apoplast, early and late xylem. To this end, fold-change values of DE genes in each condition in relation to growth in rich culture medium were used as input to the Mfuzz clustering package. Six different gene expression profile clusters were identified according to the condition or temporal progression, considering that the apoplast is the earliest stage during infection, followed by early and late xylem (Fig. 2, Additional File 7). According to this, the profile named “specific apoplast” contained 807 genes up-regulated in the apoplast but down-regulated in early and late xylem (Fig. 2a), and the profile “specific xylem” contained 1286 genes down-regulated in the apoplast but up-regulated in the other conditions (Fig. 2b). We identified two additional profiles, including genes that continuously decreased (561 genes up-regulated in the apoplast with transcripts gradually decreasing in xylem) (Fig. 2c) or increased (334 genes, opposite profile) (Fig. 2d) their expression over the infection period. Finally, the genes specifically repressed (Fig. 2e) or induced (Fig. 2f) in the early xylem that showed the opposite trend in the apoplast and late xylem were 105 and 107, respectively.

To unveil the biological functions behind each expression profile, we performed enrichment analyses. Enriched KEGG pathways in the “specific apoplast” expression profile included various biosynthetic processes, especially biosynthesis of secondary metabolites (99 out of 308 tagged genes) and related pathways such as biosynthesis of amino acids (53 genes) and flagellar assembly (25 genes) (Fig. 2a). Our manually-defined motility subcategory was enriched in this expression profile (*p-value* = 1.78·10^− 2^). In the “specific xylem” profile, the KEGG enrichment analysis yielded terms related with metabolism adaptation such as microbial metabolism in diverse environments (106 out of 411 tagged genes), ABC transporters (63 genes), and nitrogen metabolism (19 genes) among others (Fig. 2b). Our manually-defined subcategories T3SS & T3Es (*p-value* = 5.2·10^− 3^), phytohormones (*p-value* = 2.5·10^− 3^) and nitrogen metabolism (*p-value* = 2·10^− 6^) were also significantly enriched in this profile. KEGG enriched terms within the continuous decrease profile were linked to transcription and carbohydrate metabolism such as ribosome (43 out of 191 tagged genes) and carbon metabolism (24 genes) (Fig. 2c). Finally, the profile containing genes with specific up-regulation in the early xylem, was enriched in the ubiquinone and other terpenoid-quinone biosynthesis pathway (3 out of 22 tagged genes). The subcategory T3SS & T3Es was significantly enriched in this expression profile as well (*p-value* = 1.34·10^− 2^), containing genes such as the master regulator *hrpB,* and three T3 effectors (Fig. 2f). GO enrichment analysis confirmed these results, showing over-represented categories with similar biological functions (Additional File 8).

### *R. solanacearum* specifically activates different sets of virulence factors in different plant environments

**Fig. 3:**
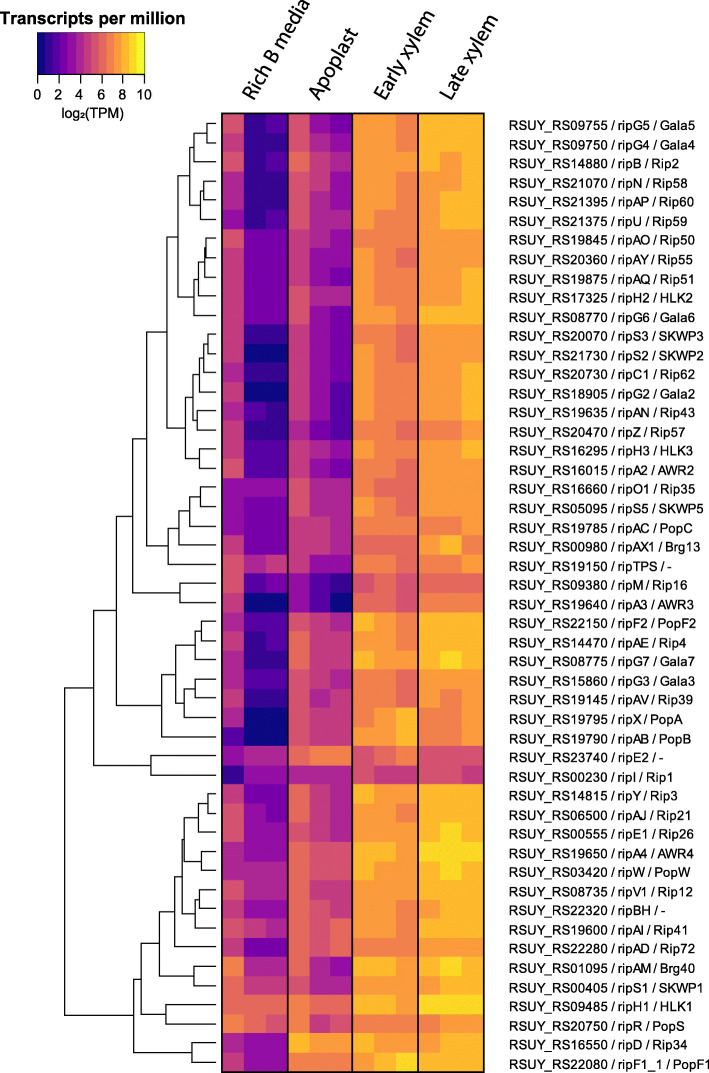
**T3E gene expression profile**. Heatmaps showing the normalised transcripts per million (TPM) of the genes coding the 60 T3E described in *R. solanacearum* UY031 in the reference condition and *in planta* apoplast, early and late conditions. Only putatively functional T3E genes are included according to Peeters et al., 2013

**Fig. 4:**
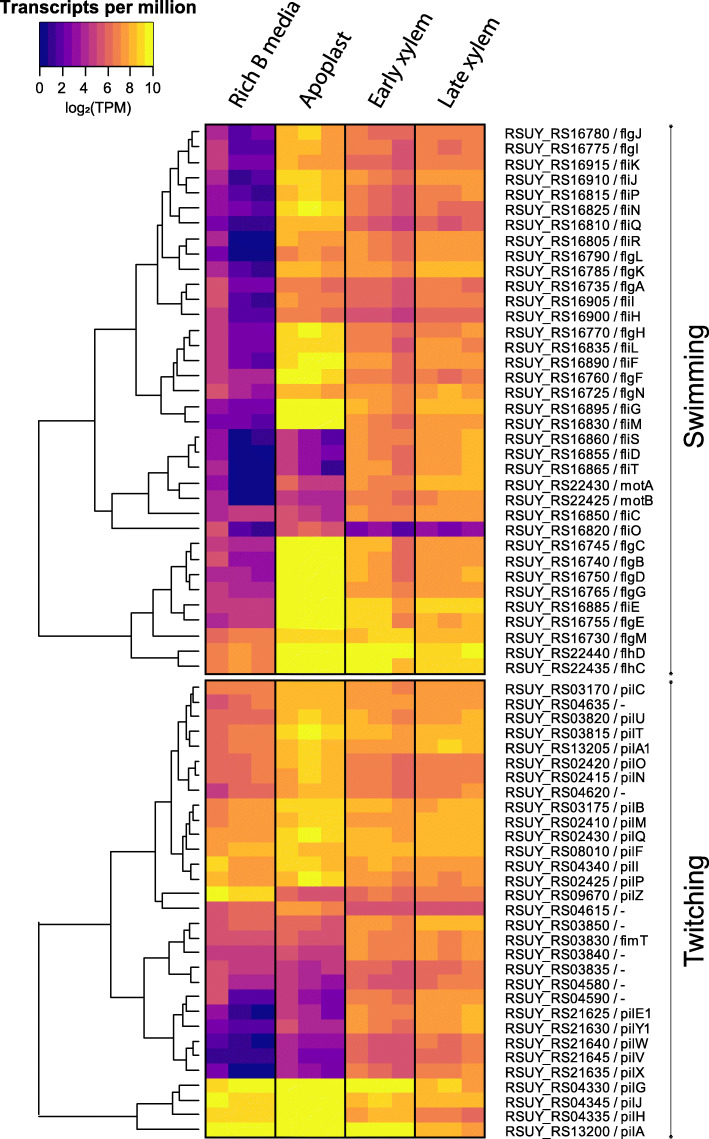
**Motility gene expression profile**. Heatmaps showing the normalised transcripts per million (TPM) of the genes involved in motility in the reference (rich Medium), apoplast, early and late xylem conditions. Two heatmaps are shown to differentiate the swimming (top panel) and twitching (bottom panel)

As described above, key virulence activities were induced in specific plant environments or at specific disease stages. To analyse in further detail the genes in this “virulence and parasitic fitness” (Additional File 6) and its subcategories we graphically represented their normalised read counts in all assayed conditions, including the reference condition in rich medium. This provided an unbiased view on the gene expression data avoiding the effect of the reference condition in the DESeq analysis. Detailed observation of gene expression values in heatmap representations for the T3SS (*hrp* and *hrc* genes) and T3E (*rip* genes) reinforced the above-described enrichment in various genetic programmes or conditions (Fig. 3, Additional File 9). Both the *rip* T3Es and the *hrp/hrc* genes displayed a very homogeneous expression pattern with high expression levels in the xylem genetic programme (early and late) and low expression levels in the apoplast. The only exceptions among the effectors were the two *ripI* genes, with low expression levels in all studied conditions, *ripE2*, with higher expression in the apoplast, and a cluster of effector genes (i.e. *ripAD* and *ripD*), showing high transcript levels in all conditions (Fig. 3). Heatmap visualisation of the normalised transcriptomic data also indicated that flagellar genes —essential for swimming motility— were highly expressed in all *in planta* conditions, but to a higher extent in the apoplast (Fig. 4 top panel). This is in accordance with the enrichment of this category *in planta* and in the late xylem genetic programmes up-regulated genes, as well as in the specific apoplast profile. The *pil* twitching motility genes encoding type IV pili followed a similar trend, although their expression was more similar in the apoplast and the xylem (Fig. 4 bottom panel), suggesting that the bacterium is using the pilus appendix in all assayed plant environments. Exceptions to this trend were the flagellar genes (i.e. *fliM, fliS, fliD, fliT, motA, motB, fliC, fliO*) and the type IV pilus genes (e.g. *pilE1, pilY1, pilW, pilV, pilX*), which were down-regulated in the apoplast compared to the xylem genetic programme. The genes encoding chemotactic sensors and chemotaxis signal transduction proteins showed low expression levels in the apoplast and progressive induction in the early and late xylem conditions (Additional File 10) in accordance with the enrichment of these specific genes in the late xylem genetic programme. Finally, all the UY031 genes that synthetize the plant hormones ethylene (*efe*), cytokinin (*tzs*), and auxin (*RSUY_RS1835* to *RSUY_RS18970*) [33] were highly expressed in the xylem genetic programme and to a lower extent in the apoplast, *efe* and *tsz* displaying a more sustained expression in the apoplast. This stable expression in all *in planta* conditions was also observed in the differential expression analysis (Additional File 12).

## Discussion

### *R. solanacearum* gene expression displays a behavioural differentiation into four plant genetic programmes that develop over time during in planta infection

Previous *R. solanacearum* transcriptomic studies compared gene expression profiles obtained using a specific *in planta* condition, such as root apoplast [36] or early xylem colonisation [14], to reference bacteria grown in rich medium. In our study, we analysed the whole infection process, including three different *in planta* conditions: apoplast, early and late xylem, which typify paradigmatic stages of infection. Intersection of the DEGs of each of the three *in planta* experimental conditions showed that most of the DEGs of *R. solanacearum* during the infection are grouped in four biologically relevant genetic programmes: genes commonly DE in all *in planta* conditions, genes exclusively DE in the apoplast, genes expressed in the xylem at any stage of the disease and genes exclusively DE in the xylem when plants are already wilted (Fig. 1). One of the previous transcriptomic studies sampled bacteria from plants 5 days post-inoculation [14], similar to our early xylem condition. With the addition of our novel late xylem condition 10 days after inoculation, and the apoplast condition, we provide a more detailed expression landscape of *R. solanacearum*, encompassing important different stages of the infection process. To study the transcriptomic data from a tissue-specific perspective, we clustered the DEGs based on their expression profile across the three *in planta* experimental conditions (i.e. apoplast, early and late xylem) (Fig. 2). Reinforcing the concept of a specific behaviour of *R. solanacearum* in different genetic programmes, the largest number of DEGs appeared exclusively up-regulated in the xylem genetic programme (1286 genes) and the apoplast (807 genes). This finding confirms that *R. solanacearum* has different sets of genes that are deployed to infect the plant and adapt to the environments encountered along the infection. It should be noted that our gene expression experiments *in planta* were all performed at comparable bacterial loads. The reason for this is that *R. solanacearum* forms microcolonies and biofilms at early infection stages in the apoplast [50], so that neither the effective local bacterial concentrations nor if density-dependent regulatory circuits are already induced at these early stages are known. Consequently, although our results perfectly reflect *R. solanacearum* adaptation to different plant environments, the influence that bacterial cell densities have on their gene expression during disease progression is not reflected in our results.

### T3Es expression is prevalent throughout the *in planta* infection process, especially in the xylem

**Fig. 5:**
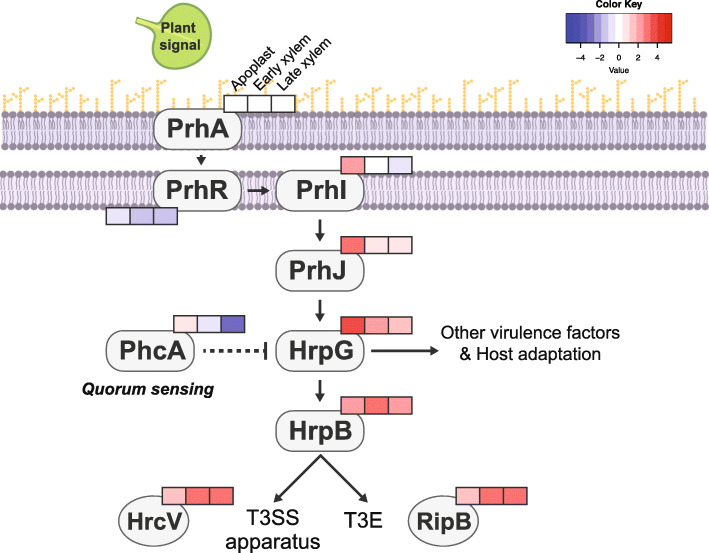
**T3SS regulatory cascade**. Log_2_ fold-change expression profile in the Apoplast, Early and Late Xylem of the genes involved in the T3SS regulatory cascade and downstream activated genes. Log_2_ fold changes in transcript levels with respect to the control condition (axenic growth in rich medium) are indicated in the boxes (left to right: Apoplast, Early and Late Xylem conditions) in colour gradients according to the Colour Key

Here, we carefully investigated the expression pattern of the most important virulence factors to elucidate the bacterial strategies used to rewire the plant environments to its own benefit. The T3SS is the main pathogenicity determinant in *R. solanacearum*, as *hrp* mutants are completely avirulent [51]. The T3SS is tightly regulated by a transcriptional regulatory cascade that contains the constitutive receptor and transducer elements PrhA and PrhR and the transcriptional regulators PrhJ, HrpG and HrpB [31]. Interestingly, in this work we found that this cascade appears sequentially induced during infection. As depicted in Fig. 5, *prhI* and *prhJ* are exclusively induced in the apoplast, *hrpG* expression also peaks in this environment but is sustained at lower levels in the early xylem and *hrpB* is expressed in the apoplast but highly induced in the early xylem, preceding the expression of the T3SS and most T3E, which is maximal at all xylem stages (Additional File 9).

Our gene expression dataset also shows that most of the 60 T3Es are highly induced in the xylem genetic programme, confirming our previous results [34] that challenged the view of T3Es as key only early after infection [32, 40]. In agreement our finding that almost all T3Es are simultaneously expressed in the xylem, a recently published study showed that deletion of 42 *R. solanacearum* T3E genes was required to compromise virulence of the bacteria on tobacco and eggplant and proliferation inside the xylem [52].

Interestingly, all *R. solanacearum* T3Es belonging to gene families (*PopA/B/C*, *AWR2/3/4/5_1/5_2*, *SKWP1/2/3/5/7*, *HLK1/2/3* and *GALA2/3/4/5/6/7*) [53] were clearly induced in the xylem throughout infection (Fig. 3). The *GALA* effectors (e.g. *ripG2 to ripG7*) and the *AWR* effectors (e.g. *ripA2 to RipA5*) have both been shown to be collectively required for full bacterial virulence and to target the proteasome or inhibit the Target Of Rapamycin (TOR) pathway, respectively [54–56]. Their expression pattern suggests that these biochemical activities are likely carried out in the xylem. Similarly, The T3E *ripAY*, which has been proven to impair the redox status of the plant cell degrading glutathione through its gamma-glutamyl cyclotransferase activity [57–60], was clearly induced in the xylem (Fig. 3). This points to the xylem as a stressing redox environment *R. solanacearum* must cope with.

In contrast, a few T3Es showed alternative induction patterns to the one described above. For instance, *ripE2* can be clearly classified as an “early effector” since it was highly induced in the apoplast compared to the other conditions, while *ripD* and *ripAD* were highly induced in all *in planta* conditions (Fig. 3). *RipD* localizes in vesicle-like structures and blocks the flg22-induced ROS response in *Nicotiana benthamiana* [61]. This fact, linked with its high expression in the apoplast and the activation of flagellar genes in this condition, suggests that *R. solanacearum* counteracts flg22 plant defence responses from the first stages of infection onwards. On the contrary, *ripI,* which was shown to enhance plant production of gamma-aminobutyric acid (GABA)*,* was lowly expressed in all *in planta* conditions (Fig. 3) [62]. Although GABA catabolization by *R. solanacearum* enhances its infection capacity, the overproduction of GABA in plant cells in the absence of sufficient bacteria to consume it has been shown to induce cell death [62]. Therefore, we hypothesize that RipI expression inside the plant must be tightly regulated to induce the production of nutrients without triggering plant stress signals.

### *R. solanacearum* modulates twitching and swimming in different plant environments

*R. solanacearum* uses two types of motility during the colonisation of plant tissues: swimming [26] and twitching [27]. Swimming motility is an individual bacterial movement through liquid environments in which flagella rotate by a proton-driven motor that is directed by chemosensor proteins [63]. Previous research showed that both flagella (*fliC*) and chemosensor (*cheA* and *cheW*) mutants were less virulent than the wild-type *R. solanacearum*, demonstrating that not just the flagellar movement but also the ability to direct it are essential for full virulence *in planta* [8]. Interestingly, full virulence was restored when the chemotactic mutants were directly inoculated in the plant stem, indicating that swimming motility is of crucial importance at the very early stages of infection [8]. In our data (Fig. 4), most of the flagellar-encoding genes were highly induced in the apoplast and, to a lower extent, in the early and late xylem, supporting the previously mentioned hypothesis. A small subset of flagellar genes including the motor (*motA, motB*), the flagellin subunit (*fliC*) and the filament cap (*fliD*) among others showed low expression in the apoplast, for which we have no plausible explanation.

*R. solanacearum* displays twitching motility, which involves the extension and retraction of type IV pili to move on solid or viscous surfaces [64]. This motility is involved in natural transformation, biofilm formation and virulence [27]. Inactivation of the genes encoding the pilin protein (*pilA*), the secretin involved in the pilus extrusion (*pilQ*) or the protein required for pilus retraction (*pilT*) reduced *R. solanacearum* virulence [65]. In our transcriptomic data, twitching motility genes showed a similar expression pattern than swimming motility, but they were less induced in the apoplast and their expression was often maintained in early and late xylem (Fig. 4). This emphasizes the importance of twitching motility throughout the plant infection process, as showed by the effect on virulence of *pil* deletion mutants [27, 65, 66]. Finally, *pilI*, which encodes the type IV pili chemosensor protein, was especially induced in the apoplast (Fig. 4 bottom panel), in agreement with our recent findings that it is involved in virulence especially during the early infection stages [66].

### *R. solanacearum* specifically activates different nitrogen metabolism genes to thrive in the xylem

**Fig. 6:**
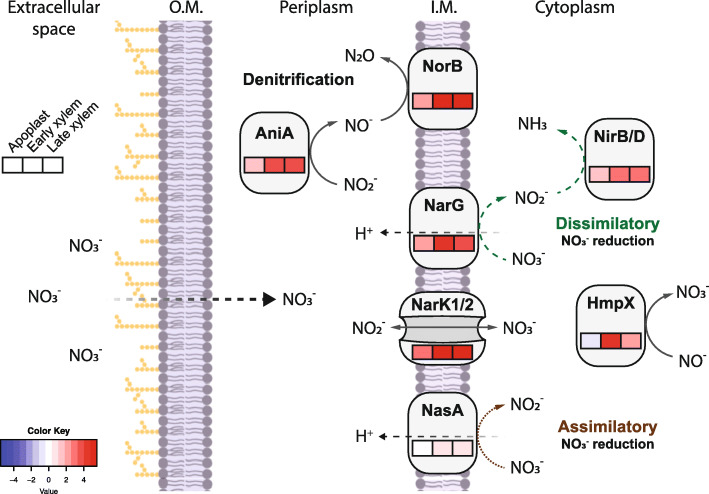
**Nitrogen metabolism expression profile**. Log_2_ fold-change expression profile in the Apoplast, Early and Late Xylem of the genes involved in the denitrification (*aniA, norB*), assimilatory (*nasA*), dissimilatory (*narK1/2, narG, nirB/D*) and nitrite detoxification (*hmpX*) pathways of nitrogen in *R. solanacearum* UY031. Log_2_ fold changes in transcript levels with respect to the control condition (axenic growth in rich medium) are indicated in the boxes (left to right: Apoplast, Early and Late Xylem conditions) in colour gradients according to the Colour Key. O. M = Outer membrane, I.M. = Inner membrane

*R. solanacearum* encounters a hypoxic environment in the plant xylem, which could limit its growth as the bacterium usually uses oxygen as the main terminal electron acceptor. However, the xylem contains an optimal concentration of nitrate that *R. solanacearum* can use as terminal electron acceptor to maintain its growth rates in this environment [29]. Our gene expression dataset shows a faint induction of the nitrogen metabolism in the apoplast, reaching its expression peak in the xylem (Fig. 6). When nitrate is available in the extracellular space, it diffuses the outer membrane and is imported to the cytoplasm by NarK1/2. Once nitrate enters the cytoplasm, the nitrate reductase (NarG/H/I) converts it to nitrite and then to ammonia through nitrite reductase (NirB/D). We found both the transporters- and reductase-encoding genes induced in the xylem (Fig. 6), suggesting that both import and dissimilatory nitrate reduction are active in this compartment (Fig. 6).

Nitrite diffusing back to the periplasm allows *R. solanacearum* to perform denitrification, first by reducing nitrite to nitric oxide via the nitrite reductase AniA and finally by reducing nitric oxide to nitrous oxide via the nitric oxide reductase NorB. Expression of these denitrification pathway genes is also induced in the xylem (Fig. 6), suggesting that *R. solanacearum* has the ability to detoxify the reactive nitrogen species produced during nitrate dissimilatory pathway in the anaerobic xylem vessels [29].

Moreover, *R. solanacearum* can also incorporate nitrogen to its central metabolism through the assimilatory nitrate reduction. The nitrate present in the cytoplasm is reduced to nitrite by NasA/B. A previous study showed that nitrate assimilation was essential for initial root attachment but was dispensable for growth, virulence, and competitive fitness [28]. The fact that *nasA* is induced in the xylem and not in the apoplast is in disagreement with these results and may indicate strain- or condition-specific roles of N genes in *R. solanacearum*. Finally, the nitric oxide anion in the cytoplasm can be detoxified using HmpX, whose expression is also highly induced in the xylem genetic programme (Fig. 6), an indicator of a highly active N metabolism in this plant environment.

### Phytohormone and ROS scavenging enzymes are expressed along the infection

*R. solanacearum* genome codes for phytohormone biosynthetic genes that drive the production of auxin [33], cytokinin [67] and ethylene [68]. Interestingly, bacterially-produced auxin was described to block plant defences against the plant pathogen *Pseudomonas syringae* pv *savastanoi* [69] and ethylene was involved in wilting development in the pathosystem *A. thaliana-R. solanacearum* [70]. In this study, we observed induction of the cytokinin (*tzs*) and the ethylene (*efe*) biosynthetic genes as well as the auxin operon in the xylem (Additional File 11). Apoplastic induction of the master regulator *hrpG*, which also controls auxin and ethylene synthesis genes [33], precedes the xylematic expression of phytohormone biosynthesis genes as was observed for the T3SS (Fig. 5).

After pathogen infection, plant cells respond with ROS production to create a hostile environment against the bacterium [71]. Interestingly, *R. solanacearum* contains several genes that code for ROS scavenging enzymes, helping the bacterium survive in the plant apoplast and xylem [72]. Amongst them, alkyl hydroperoxide reductase genes (*ahpC1/C2/D/F*) were mostly induced in the xylem (Additional File 12). Several studies have linked the induction of *ahp* genes in biofilm-forming cells in different bacterial pathogens, contributing to protection against oxidative stress, epiphytic survival and attachment in the intercellular spaces or to the xylem vessels [73–77].

## Conclusion

In summary, we performed a transcriptomic analysis of *R. solanacearum* at different conditions in potato plants. DEG analysis revealed that *R. solanacearum* deploys inside the plant host four different genetic programmes. Functional enrichment analysis showed that *R. solanacearum* has the highest expression of motility genes in the apoplast, while the majority of T3Es and nitrogen metabolism genes are highly induced in the xylem environment. This study provides for the first time a dynamic gene expression landscape of the bacterial plant pathogen *R. solanacearum* and is a first step towards the transcriptomic characterisation of its complete infection cycle.

## Methods

### Bacterial strains and plant growth conditions

The highly aggressive *Ralstonia solanacearum* strain UY031 (phylotype IIB, sequevar 1) isolated from potato tubers in Uruguay [46] carrying the synthetic *luxCDABE* operon under the control of the *psbA* promoter was used in this study [34]. The luminescence allowed indirect but precise quantification of bacteria and to track bacterial proliferation *in planta* [48]. Bacteria were routinely grown at 30 °C in rich B medium supplemented with 0.5% glucose [34].

*Solanum tuberosum* cv. Desirée potato plants were propagated in vitro [36] and 2-week old apexes were transferred to a soil:silica sand mixture in a 1:1 ratio for RNA-seq sampling or moved to a substrate:perlite:vermiculite mixture in a 30:1:1 ratio for *in planta* visualisation. Plants were grown at 22 °C under long day (16 h / 8 h light/dark) conditions for 3 weeks.

### Bacterial sampling

For liquid medium samples, bacterial cultures were set to an starting OD_600_ = 0.1 (10^8^ CFU/ml) and grown for 5 h in rich B medium (10 g/L bacteriological peptone, 1 g/L yeast extract, 1 g/L casamino acids), until they reached exponential growth phase (OD_600_ ~ 0.4–0.5). Bacteria were then centrifuged at 4 °C for 2 min at maximum speed and the pellet was immediately frozen in liquid nitrogen.

To assess bacterial colonisation levels, especially in asymptomatic plants, stems were placed under a luminometer to visualize bacterial densities within the vascular system, and only plants showing luminescence were used. To avoid bias of quorum sensing signals in the xylem stages and not in the apoplast, similar bacterial yields were infiltrated in potato leaves for the initial stage. Finally, to identify the best time point at which bacterial colonisation within xylem vessels of almost asymptomatic plants was most similar to that in dead plants, we monitored bacterial growth, luminescence and disease symptoms over time (Additional File 1 A). As shown in Additional File 2 A, bacterial densities recovered from the three *in planta* conditions were in the same order of magnitude (between 10^7^ and 10^8^ CFUs/ml). The in vitro reference condition corresponding to bacteria grown in liquid rich medium, was also obtained to better define *R. solanacearum* gene expression. We ensured that the difference of the final bacterial yields from the different conditions was not higher than one log (Additional File 2 A). These conditions allowed us to obtain enough *R. solanacearum* RNA-seq reads to have a robust representation of the whole genome (Additional File 13). Principal component analysis revealed that these conditions are consistent among biological replicates and sensitive enough to detect biological differences between conditions (Additional File 2 C).

To obtain more reproducible samples, leaf apoplast was used as a mimic condition of root apoplast, since it has been reported that *R. solanacearum* behaves similarly in these two apoplastic spaces [47].

To obtain leaf apoplast samples, bacterial cells from an overnight culture were washed with water and resuspended to a final concentration of 5 × 10^8^ CFU/ml. The aerial part of the plants was vacuum-infiltrated for 30 s to 1 min and the leaves were dried in paper towel before incubating the plants in the inoculation chamber (27 °C, 12 h / 12 h). After 6 h, leaves were vacuum-infiltrated with sterile distilled water, dried in paper towel, rolled in a cut tip and centrifuged inside a 50 ml tube at 4 °C for 5 min at 2000 rpm. Apoplast fluid extract was pooled (each pool representing approximately 15 plants) and centrifuged at 4 °C at maximum speed for 2 min. Bacterial pellets were frozen in liquid nitrogen.

For early and late xylem samples, potato roots were injured with a 1 ml tip before inoculation. A total of 40 ml of a 10^8^ CFU/ml *R. solanacearum* suspension was used to soil-inoculate each plant. After inoculation, plants were kept inside the inoculation chamber (27 °C, 12 h / 12 h) for 6 days (mean disease index = 0–1) for early xylem condition, or 10 days (disease index = 4 in all the plants) for late xylem condition. Plants were photographed in a Fuji Film LAS4000 light imager system to check individual infection levels and only plants showing luminescence were used. Stem pieces of 2 cm were cut from each plant, placed in a 1.5 ml tube containing 500 μl of sterile distilled water and centrifuged 2 min at maximum speed at 4 °C to release bacteria from the xylem vessels. In all cases, bacterial densities were measured by luminescence before freezing and dilutions were plated to measure CFUs before addition of 5% of an ice-cold transcriptional stop solution (5% [vol/vol] water saturated phenol in ethanol). This enabled normalisation of early or late xylem samples for bacterial concentrations comparable to those of apoplast and reference medium samples. Bacterial pellets were pooled together for each biological replicate and frozen in liquid nitrogen. Approximately 30 plants were used for each early xylem replicate and 7 plants for every late xylem replicate (Additional File 2 A).

### RNA extraction, sequencing and library preparation

Total RNA was extracted using the SV Total RNA Isolation System kit (Promega) following manufacturer’s instructions for Gram-negative Bacteria. RNA concentration was measured with a ND-8000 Nanodrop and RNA integrity was validated for all samples using the Agilent 2100 Bioanalyzer. For rRNA depletion, 2.5 μg of total RNA were treated with the Ribo-zero (TM) magnetic kit for bacteria (Epicentre). Three biological replicates per condition were subjected to sequencing on a HiSeq2000 Illumina System apparatus using multiplexing and kits specially adapted to obtain 100 bp paired-end reads in stranded libraries. Rich media reference samples were sequenced by Macrogen Inc. In all other cases, RNA-sequencing was performed in the Shanghai PSC Genomics facility. Raw sequencing data will be available upon publication in the Sequence Read Archive under Bio Project: PRJNA660623 (accession codes SAMN15955133 to SAMN15955144).

### Read alignment, mapping and differential gene expression analysis

RNA-seq raw data quality was evaluated using FASTQC (version 0.11.4, [78]). *R. solanacearum* reads were mapped using Bowtie2 (version 2.3.3, [79]) with stringent parameters [36] using as reference the completely sequenced genome of UY031 strain [80]. Alignment files were quantified with HTSeq-count (version 0.11.3, [81]) using NCBI’s RefSeq sequences NZ_CP012687.1 (chromosome) and NZ_CP012688.1 (megaplasmid). The DESeq2 package (version 1.28.1, [82]) in R ( [83], ver. 3.6.3) was employed to perform differential expression (DE) analysis of high quality RNAseq reads. Genes with |log_2_(fold-change) | > 1.5 and adjusted *p-value* < 0.01 were considered as DE *in planta* when compared to bacteria grown on liquid rich medium as reference condition (Additional File 2 C). The results of the DeSeq2 analysis is shown in Additional File 3. The UpSetR [84] R package was used to visualise the intersection of DE genes in the different *in planta* conditions. For gene expression comparison, gene counts were also normalised to transcripts per million (TPM) (Additional File 14).

### Gene expression pattern clustering and enrichment analysis

To obtain expression profiles of *R. solanacearum* UY031 genes, a soft clustering analysis was performed using Mfuzz package (version 2.48, [85]) in R. Input data corresponds to the DE fold-change values yielded by DESeq2 of apoplast, early and late xylem samples normalised to the reference liquid rich medium. The cluster number was manually set at c = 6. To be more stringent, a gene was considered to belong to a specific cluster if the gene was allocated in the same cluster in 30 out of 40 iterations with the membership value set to μ ≥ 0.75.

To further characterise the genes differentially expressed or belonging to any of the clusters, we looked for enriched Gene Ontology (GO) terms or Kyoto Encyclopaedia of Genes and Genomes (KEGG) pathways among our genes. Since no GO terms had been previously associated to UY031 strain genes, we used Blast2GO [86] software to annotate the UY031 genome. For the KEGG and GO enrichment analysis, we used the enricher function of the ClusterProfiler package [87] in R having previously created the TERM2GENE and TERM2NAME lists to do the hypergeometric test.

Because KEGG enrichment analysis is limited to a number of pre-established pathways or terms that do not include important virulence categories, and because pathogenicity-related terms in GO are too general and have not been widely used, we decided to create a manually curated category that we defined as “virulence and parasitic fitness (Additional File 6). This category included the T3SS and type III effectors, motility genes, exopolysaccharides secretion, phytohormone biosynthesis, ROS scavenging, nitrogen metabolism and cell-wall degrading enzymes. After defining the genes included in this category, we conducted a hypergeometric test using the R stats package on the differentially expressed genes or the gene clusters to find out whether the “virulence and parasitic fitness” or any of the subcategories was overrepresented.

### In planta visualisation of *R. solanacearum*

To visualize *R. solanacearum* bacterial cells in early (6 days post-inoculation, d.p.i) and late (10 d.p.i.) xylem stages, UY031 with the *psbA* constitutive promoter was fused to the GFP gene. This reporter strain was soil-inoculated with root wounding at OD_600_ = 0.1 (10^8^ CFUs/ml) in 3 week-old potato plants. Potato stem slices from the first node of infected plants with GFP-containing bacteria were observed in the SZX16 stereomicroscope equipped with a DP71 camera system (Olympus). Pictures were obtained using the following settings: GFP filter, 10 s exposure time, ISO 1/800. Control plants were soil-inoculated with water (Additional File 1 B).

## Supplementary Information

**Additional file 1:** *R. solanacearum* reporter strains and bacterial growth show equivalent infection rates. (A) Luminescence levels or bacterial growth (bar plot) and symptom development (line plot) in potato plants were monitored over time to detect the precise time points at which similar bacterial yields but different symptoms could be detected. The disease index scale (DI) ranges from 0 to 4 being 0 symptomless plants and 4 plants completely wilted. Luminescence measurement were conducted on stem sections of infected plants. (B) GFP-labelled bacteria were monitored at the sampled time points in potato plants. RLU = Relative light units.

**Additional file 2:** RNAseq experimental set-up and bioinformatic pipeline. (A) Experimental set-up for the three *in planta* conditions, corresponding to an early (leaf apoplast), mid (xylem from asymptomatic plants) and late stages (xylem from dead plants) of the disease. As reference condition, bacteria grown in rich liquid media were used. The average of bacterial yields recovered in each condition are indicated as CFU/ml. The grey background section of the figure contains the representation of how bacteria was enriched in each condition (see M&Ms). (B) Transcriptomic analysis pipeline. (C) Two-dimensional Principal Component Analysis representation of the expression data of the conditions’ biological replicates used in the study.

**Additional file 3:** DEGs in the three *in planta* conditions. Differentially expressed genes of *R. solanacearum* in apoplast, early and late xylem compared to liquid rich medium obtained with DESeq2 (*p-adj* > 0.01, log_2_ FC ± 1.5).

**Additional file 4:** Overlap of DEGs in apoplast, early and late xylem compared to previous gene expression analysis. (A) Percentage of common DE genes in each *in planta* condition (versus rich medium) compared to previous *in planta* gene expression analyses (− Puigvert et al. 2017; −Jacobs et al. 2012; −Khokhani et al. 2017). Fractions represent the overlapping genes from the total of DEGs in each of our conditions compared to a given previous gene expression analysis. Colors were plotted using the Conditional Formatting tool in Microsoft Excel. (B) Expression correlation of the DE data of the common genes between our Apoplast data and the RNAseq data from the potato root (Puigvert et al. 20,107).

**Additional file 5:** Transcriptomic profile of *R. solanacearum* in *in planta* genetic programmes. Up-regulated (left) and down-regulated (right) genes shared and unique across the three *in planta* conditions. Each vertical bar plot represents the number of shared DE between the conditions indicated by the lines and dots in the schematic below. The horizontal bar plots on the right indicate the total DE genes per *in planta* condition compared to rich medium. For the intersection of Apoplast, Early and Late (*in planta* environment), Early and Late (Xylem environment), Apoplast and Late xylem alone, the list of genes was extracted and surveyed for enriched GO terms. Dot plots of the enriched GO terms for the up- (left) and down-regulated (right) genes in each environment is shown below. DE genes were identified with DEseq2 (*p-adj* > 0.01, log_2_ FC ± 1.5) and plotted using the R package UpsetR.

**Additional file 6:** “Virulence and parasitic fitness” manually defined category. Genes belonging to specific virulence categories (T3SS & T3Es, Motility, ROS scavenging enzymes, phytohormone biosynthesis, EPS, nitrogen metabolism, cell wall degrading enzymes) of *R. solanacearum* are listed showing information related to: UY031 NCBI locus tag (first column), gene name (second column), gene description (third column), category, (forth column), reference (fifth column).

**Additional file 7:** List of genes included in each of the six expression profiles.

**Additional file 8:** Gene expression dynamics of *R. solanacearum* throughout infection. Six clusters were obtained through Mfuzz clustering of log_2_- fold-change data of the apoplast, early and late xylem conditions normalised to the reference rich liquid media. Clusters include the genes (number indicated above each graph) with a membership higher than 70% and consistently associated to the same cluster on at least 30 out of 40 iterations. The list of genes associated to each cluster was extracted and surveyed for enriched GO terms. Dot plots of the enriched GO terms in each cluster is shown next to the cluster.

**Additional file 9:** T3SS regulatory cascade and apparatus gene expression profile. Heatmap showing the normalised transcripts per million (TPM) of the genes involved in the T3SS regulatory cascade and the T3SS apparatus in the reference and in the *in planta* conditions.

**Additional file 10:** hemosensors and signal transduction gene expression profile. Heatmap showing the normalised transcripts per million (TPM) of the genes involved in chemosensing and signal transduction in the reference and in the *in planta* conditions.

**Additional file 11:** Phytohormones biosynthesis gene expression profile. Heatmap showing the normalised transcripts per million (TPM) of the genes involved in phytohormones biosynthesis in the reference and in the *in planta* conditions.

**Additional file 12:** ROS scavenging enzymes gene expression profile. Heatmap showing the normalised transcripts per million (TPM) of the genes coding for ROS scavenging enzymes in the reference condition and *in planta* apoplast, early and late condition.

**Additional file 13:** Proportion of reads aligned to *R. solanacearum* UY031 genome. Total number of reads obtained in each biological replicate for each condition (first column). Total number of reads aligned to *R. solanacearum* UY031 genome (second column). Proportion of reads aligned to *R. solanacearum* genome expressed as percentage (third column).

**Additional file 14:** Transcripts Per Million of each gene in rich medium, apoplast, early and late xylem. Reads normalized per Transcripts Per Million for each *R. solanacearum* gene in every condition: rich medium (philiq1, 2, 3), apoplast (Apo.10, .7, .9), early xylem (Early.D, .E, .G) and late xylem (Xylem.E, .O, Fresh.xylem). (CSV 986 kb)

## Data Availability

All data generated or analysed during this study are included in this published article and its supplementary information files and the supporting datasets are available in Sequence Read Archive repository under Bio Project: PRJNA660623 (accession codes SAMN15955133 to SAMN15955144) in https://www.ncbi.nlm.nih.gov/sra.

## Abbreviations

CFU: Colony forming units
Cv: Cultivar
d.p.i.: Days post inoculation
DEG: Differentially Expressed Genes
DI: Disease index
DNA: desoxyribonucleic acid
EPS: exopolysaccharide
GABA: gamma-aminobutyric acid
GFP: Green Fluorescent Protein
GO: Gene Ontology
*Hrp*: Hypersensitive response and pathogenicity
IVET: In vivo expression technology
KEGG: Kyoto Encyclopedia of Genes and Genomes
OD: Optical Density
PCA: Principal component analysis
RLU: Relative Light Unit
RNA: Ribonucleic acid
ROS: Reactive Oxygen Species
rRNA: Ribosomal ribonucleic acid
T3E: Type 3 Effector
T3SS: Type III Secretion System
TCA: Tricarboxylic acid

## References

[1] Coll NS, Valls M. Current knowledge on the Ralstonia solanacearum type III secretion system. Microb Biotechnol [Internet]. 2013 Nov [cited 2020 Sep 18];6(6):614–20. Available from: https://www.ncbi.nlm.nih.gov/pmc/articles/PMC3815929/10.1111/1751-7915.12056 PMC3815929 23617636

[2] Peeters N, Guidot A, Vailleau F, Valls M. Ralstonia solanacearum, a widespread bacterial plant pathogen in the post-genomic era. Mol Plant Pathol [Internet]. 2013 Sep 1 [cited 2020 Sep 18];14(7):651–62. Available from: https://bsppjournals.onlinelibrary.wiley.com/doi/full/10.1111/mpp.12038 10.1111/mpp.12038 PMC6638647 23718203

[3] Hayward AC (1991). Bacterial wilt caused by pseudomonas solanacearum. Annu Rev Phytopathol.

[4] Ciampi L, Sequeira L, French ER (1980). Latent infection of potato tubers by Pseudomonas solanacearum. Am Potato J.

[5] Janse JD, Van Den Beld HE, Elphinstone J, Simpkins S, Tjou-Tam-Sin NNA, Van Vaerenbergh J (2004). Introduction to Europe of Ralstonia solanacearum biovar 2, race 3 in Pelargonium zonale cuttings. J Plant Pathol.

[6] Champoiseau PG, Jones JB, Allen C (2009). Ralstonia solanacearum race 3 Biovar 2 causes tropical losses and temperate anxieties. Plant Heal Prog.

[7] Álvarez B, López MM, Biosca EG (2007). Influence of native microbiota on survival of Ralstonia solanacearum phylotype II in river water microcosms. Appl Environ Microbiol.

[8] Yao J, Allen C (2006). Chemotaxis is required for virulence and competitive fitness of the bacterial wilt pathogen Ralstonia solanacearum. J Bacteriol.

[9] Vasse J, Frey P, Trigalet A (1995). Microscopic studies of intercellular infection and protoxylem invasion of tomato roots by Pseudomonas solanacearum. Mol Plant-Microbe Interact.

[10] Du Y, Stegmann M, Misas-Villamil JC (2015). Meetings the apoplast as battleground for plant – microbe interactions.

[11] Hikichi Y, Yoshimochi T, Tsujimoto S, Shinohara R, Nakaho K, Kanda A (2007). Global regulation of pathogenicity mechanism of Ralstonia solanacearum. Plant Biotechnol.

[12] Planas-Marquès M, Bernardo-Faura M, Paulus J, Kaschani F, Kaiser M, Valls M, et al. Protease Activities Triggered by Ralstonia solanacearum Infection in Susceptible and Tolerant Tomato Lines. Mol Cell Proteomics [Internet]. 2018 Jun 1 [cited 2020 Sep 18];17(6):1112–25. Available from: https://pubmed.ncbi.nlm.nih.gov/29523767/10.1074/mcp.RA117.000052 PMC5986253 29523767

[13] Zuluaga AP, Puigvert M, Valls M. Novel plant inputs influencing Ralstonia solanacearum during infection. Front Microbiol [Internet]. 2013 Nov 20 [cited 2020 Sep 18];4(NOV):349. Available from: http://journal.frontiersin.org/article/10.3389/fmicb.2013.00349/abstract 10.3389/fmicb.2013.00349 PMC3834233 24312090

[14] Jacobs JM, Babujee L, Meng F, Physiological C, Strategies V, Wilt B (2012). The In Planta Transcriptome of Ralstonia solanacearum. Conserved.

[15] Planas-Marquès M, Kressin JP, Kashyap A, Panthee DR, Louws FJ, Coll NS, et al. Four bottlenecks restrict colonization and invasion by the pathogen Ralstonia solanacearum in resistant tomato. J Exp Bot [Internet]. 2020 Mar 1 [cited 2020 Sep 18];71(6):2157–71. Available from: https://academic.oup.com/jxb/article/71/6/2157/5686149 10.1093/jxb/erz562 PMC7242079 32211785

[16] Genin S (2010). Molecular traits controlling host range and adaptation to plants in Ralstonia solanacearum. New Phytol.

[17] Vasse J, Genin S, Frey P, Boucher C, Brito B. The hrpB and hrpG regulatory genes of Ralstonia solanacearum are required for different stages of the tomato root infection process. Mol Plant-Microbe Interact. 2000; 10.1094/MPMI.2000.13.3.259 10707351

[18] Lu H, Lema SA, Planas-Marquès M, Alonso-Díaz A, Valls M, Coll NS (2018). Type III secretion-dependent and-independent phenotypes caused by Ralstonia solanacearum in Arabidopsis roots. Mol Plant-Microbe Interact.

[19] Büttner D, He SY (2009). Type III protein secretion in plant pathogenic bacteria. Plant Physiol.

[20] Coburn B, Sekirov I, Finlay BB (2007). Type III secretion systems and disease. Clin Microbiol Rev.

[21] Peeters N, Carrère S, Anisimova M, Plener L, Cazalé AC, Genin S (2013). Repertoire, unified nomenclature and evolution of the Type III effector gene set in the Ralstonia solanacearum species complex. BMC Genomics.

[22] Prior P (2013). 1998.

[23] Huang J, Denny TP, Schell MA (1993). VsrB, a regulator of virulence genes of Pseudomonas solanacearum, is homologous to sensors of the two-component regulator family. J Bacteriol.

[24] Yaowei K, Jianzhong H, Mao G, Yuan HL, Schell MA (1994). Dramatically reduced virulence of mutants of Pseudomonas solanacearum defective in export of extracellular proteins across the outer membrane. Mol Plant-Microbe Interact.

[25] Liu H, Zhang S, Schell MA, Denny TP (2005). Pyramiding unmarked deletions in Ralstonia solanacearum shows that secreted proteins in addition to plant cell-wall-degrading enzymes contribute to virulence. Mol Plant-Microbe Interact.

[26] Tans-Kersten J, Brown D, Allen C (2004). Swimming motility, a virulence trait of Ralstonia solanacearum, is regulated by FlhDC and the plant host environment. Mol Plant-Microbe Interact.

[27] Kang Y, Liu H, Genin S, Schell MA, Denny TP (2002). *Ralstonia solanacearum* requires type 4 pili to adhere to multiple surfaces and for natural transformation and virulence. Mol Microbiol.

[28] Dalsing BL, Allen C (2014). Nitrate assimilation contributes to Ralstonia solanacearum root attachment, stem colonization, and virulence. J Bacteriol.

[29] Dalsing BL, Truchon AN, Gonzalez-Orta ET, Milling AS, Allen C (2015). Ralstonia solanacearum uses inorganic nitrogen metabolism for virulence, ATP production, and detoxification in the oxygen-limited host xylem environment. MBio..

[30] Pegg GF (1985). Life in a black hole — the micro-environment of the vascular pathogen. Trans Br Mycol Soc.

[31] Brito B, Marenda M, Barberis P, Boucher C, Genin S (1999). PrhJ and hrpG, two new components of the plant signal-dependent regulatory cascade controlled by PrhA in Ralstonia solanacearum. Mol Microbiol.

[32] Genin S, Brito B, Denny TP, Boucher C (2005). Control of the Ralstonia solanacearum type III secretion system (Hrp) genes by the global virulence regulator PhcA. FEBS Lett.

[33] Valls M, Genin S, Boucher C (2006). Integrated regulation of the type III secretion system and other virulence determinants in Ralstonia solanacearum. PLoS Pathog.

[34] Monteiro F, Genin S, van Dijk I, Valls M (2012). A luminescent reporter evidences active expression of Ralstonia solanacearum type III secretion system genes throughout plant infection. Microbiol (United Kingdom).

[35] Ailloud F, Lowe TM, Robène I, Cruveiller S, Allen C, Prior P (2016). In planta comparative transcriptomics of host-adapted strains of Ralstonia solanacearum. Peer J.

[36] Puigvert M, Guarischi-Sousa R, Zuluaga P, Coll NS, Macho AP, Setubal JC, et al. Transcriptomes of ralstonia solanacearum during root colonization of solanum commersonii. Front Plant Sci. 2017;8. 10.3389/fpls.2017.00370 PMC5357869 28373879

[37] Garg RP, Huang J, Yindeeyoungyeon W, Denny TP, Schell MA (2000). Multicomponent transcriptional regulation at the complex promoter of the exopolysaccharide I biosynthetic operon of Ralstonia solanacearum. J Bacteriol.

[38] Clough SJ, Flavier AB, Schell MA, Denny TP (1997). Differential expression of virulence genes and motility in Ralstonia (Pseudomonas) solanacearum during exponential growth. Appl Environ Microbiol.

[39] Huang J, Carney BF, Denny TP, Weissinger AK, Schell MA (1995). A complex network regulates expression of eps and other virulence genes of Pseudomonas solanacearum. J Bacteriol.

[40] Yoshimochi T, Hikichi Y, Kiba A, Ohnishi K (2009). The global virulence regulator PhcA negatively controls the Ralstonia solanacearum hrp regulatory cascade by repressing expression of the PrhIR signaling proteins. J Bacteriol.

[41] Meng F, Babujee L, Jacobs JM, Allen C (2015). Comparative transcriptome analysis reveals cool virulence factors of Ralstonia solanacearum race 3 biovar 2. PLoS One.

[42] Khokhani D, Lowe-Power TM, Tran TM, Allen C (2017). A single regulator mediates strategic switching between attachment/spread and growth/virulence in the plant pathogen Ralstonia solanacearum. MBio.

[43] Yu X, Lund SP, Scott RA, Greenwald JW, Records AH, Nettleton D (2013). Transcriptional responses of Pseudomonas syringae to growth in epiphytic versus apoplastic leaf sites. Proc Natl Acad Sci U S A.

[44] Nobori T, Velásquez AC, Wu J, Kvitko BH, Kremer JM, Wang Y (2018). Transcriptome landscape of a bacterial pathogen under plant immunity. Proc Natl Acad Sci U S A.

[45] Lovelace AH, Smith A, Kvitko BH (2018). Pattern-triggered immunity alters the transcriptional regulation of virulence-associated genes and induces the sulfur starvation response in pseudomonas syringae pv. Tomato DC3000. Mol Plant-Microbe Interact.

[46] Siri MI, Sanabria A, Pianzzola MJ (2011). Genetic diversity and aggressiveness of Ralstonia solanacearum strains causing bacterial wilt of potato in Uruguay. Plant Dis.

[47] Hikichi Y. Interactions between plant pathogenic bacteria and host plants during the establishment of susceptibility [Internet]. Vol. 82, Journal of General Plant Pathology. Springer Tokyo; 2016 [cited 2020 Aug 18]. p. 326–31. Available from: https://link.springer.com/article/10.1007/s10327-016-0680-9

[48] Cruz APZ, Ferreira V, Pianzzola MJ, Siri MI, Coll NS, Valls M (2014). A novel, sensitive method to evaluate potato germplasm for bacterial wilt resistance using a luminescent ralstonia solanacearum reporter strain. Mol Plant-Microbe Interact.

[49] Arlat M, Gough CL, Zischek C, Barberis PA, Trigalet A, Boucher CA. Transcriptional organization and expression of the large hrp gene cluster of Pseudomonas solanacearum. Molecular plant-microbe interactions : MPMI. 1992;5:187–93. 10.1094/mpmi-5-187 1617200

[50] Mori Y, Inoue K, Ikeda K, Nakayashiki H, Higashimoto C, Ohnishi K, et al. The vascular plant-pathogenic bacterium Ralstonia solanacearum produces biofilms required for its virulence on the surfaces of tomato cells adjacent to intercellular spaces. Mol Plant Pathol. 2016. 10.1111/mpp.12335 PMC6638453 26609568

[51] Boucher CA, Barberis PA, Trigalet AP, Demery DA (1985). Transposon mutagenesis of Pseudomonas solanacearum: isolation of Tn5-induced avirulent mutants. J Gen Microbiol.

[52] Lei N, Chen L, Kiba A, Hikichi Y, Zhang Y, Ohnishi K (2020). Super-Multiple Deletion Analysis of Type III Effectors in Ralstonia solanacearum OE1–1 for Full Virulence Toward Host Plants Bacterial Strains and Culture Conditions.

[53] Poueymiro M, Genin S (2009). Secreted proteins from Ralstonia solanacearum: a hundred tricks to kill a plant. Curr Opin Microbiol.

[54] Remigi P, Anisimova M, Guidot A, Genin S, Peeters N (2011). Functional diversification of the GALA type III effector family contributes to Ralstonia solanacearum adaptation on different plant hosts. New Phytol.

[55] Solé M, Popa C, Mith O, Sohn KH, Jones JDG, Deslandes L (2012). Type III effectors displaying virulence and Avirulence activities. Mol Plant-Microbe Interact.

[56] Popa C, Li L, Gil S, Tatjer L, Hashii K, Tabuchi M, et al. The effector AWR5 from the plant pathogen Ralstonia solanacearum is an inhibitor of the TOR signalling pathway. Sci Rep. 2016. 10.1038/srep27058 PMC4891724 27257085

[57] Sang Y, Wang Y, Ni H, Cazalé AC, She YM, Peeters N (2018). The ralstonia solanacearum type III effector ripay targets plant redox regulators to suppress immune responses. Mol Plant Pathol.

[58] Wei Y, Sang Y, Macho AP (2017). The ralstonia solanacearum type III effector RipAY is phosphorylated in plant cells to modulate its enzymatic activity. Front Plant Sci.

[59] Mukaihara T, Hatanaka T, Nakano M, Oda K. Ralstonia solanacearum type III effector RipAY is a glutathione-degrading enzyme that is activated by plant cytosolic thioredoxins and suppresses plant immunity. MBio [Internet]. 2016 Apr 12 [cited 2020 Aug 18];7(2). Available from: https://pubmed.ncbi.nlm.nih.gov/27073091/10.1128/mBio.00359-16 PMC4959522 27073091

[60] Fujiwara S, Kawazoe T, Ohnishi K, Kitagawa T, Popa C, Valls M, et al. RipAY, a Plant Pathogen Effector Protein, Exhibits Robust γ-Glutamyl Cyclotransferase Activity When Stimulated by Eukaryotic Thioredoxins. J Biol Chem [Internet]. 2016 Mar 25 [cited 2020 Aug 18];291(13):6813–30. Available from: https://pubmed.ncbi.nlm.nih.gov/26823466/10.1074/jbc.M115.678953 PMC4807269 26823466

[61] Jeon H, Kim W, Kim B, Lee S, Jayaraman J, Jung G, et al. Erratum: Ralstonia solanacearum Type III effectors with predicted nuclear localization signal localize to various cell compartments and modulate immune responses in Nicotiana spp. (Plant Pathol. J., (2020) 36(1), (43–53), 10.5423/PPJ.OA.08.2019.0227). Plant Pathol J. 2020;36(3):303. 10.5423/PPJ.OA.08.2019.0227 PMC7012579 32089660

[62] Xian L, Yu G, Wei Y, Rufian JS, Li Y, Zhuang H, et al. A Bacterial Effector Protein Hijacks Plant Metabolism to Support Pathogen Nutrition. Cell Host Microbe [Internet]. 2020 Jul 30 [cited 2020 Aug 18];1–10. Available from: 10.1016/j.chom.2020.07.003. 10.1016/j.chom.2020.07.003 32735848

[63] Sampedro I, Parales RE, Krell T, Hill JE (2015). Pseudomonas chemotaxis. FEMS Microbiol Rev.

[64] Mattick JS. Type IV pili and twitching motility. Annu Rev Microbiol 2002;56:289–314. 10.1146/annurev.micro.56.012302.160938 12142488

[65] Liu H, Kang Y, Genin S, Schell MA, Denny TP (2001). Twitching motility of Ralstonia solanacearum requires a type IV pilus system. Microbiology..

[66] Corral J, Sebastià P, Coll NS, Barbé J, Aranda J, Valls M (2020). Twitching and Swimming Motility Play a Role in Ralstonia solanacearum Pathogenicity. mSphere.

[67] Genin S, Denny TP (2012). Pathogenomics of the ralstonia solanacearum species complex. Annu Rev Phytopathol.

[68] Freebairn HT, Buddenhagen IW. Ethylene production by Pseudomonas solanacearum. Nature. 1964;313(314). 10.1038/202313a0 14167811

[69] Robinette D, Matthysse AG (1990). Inhibition by agrobacterium tumefaciens and Pseudomonas savastanoi of development of the hypersensitive response elicited by Pseudomonas syringae pv. Phaseolicola. J Bacteriol.

[70] Hirsch J, Deslandes L, Feng DX, Balagué C, Marco Y (2002). Delayed symptom development in ein2-1, an Arabidopsis ethylene-insensitive mutant, in response to bacterial wilt caused by Ralstonia solanacearum. Phytopathology..

[71] Bolwell GP (2002). The apoplastic oxidative burst in response to biotic stress in plants: a three-component system. J Exp Bot.

[72] Flores-Cruz Z, Allen C (2009). Ralstonia solanacearum encounters an oxidative environment during tomato infection. Mol Plant-Microbe Interact.

[73] Büttner D, Bonas U (2010). Regulation and secretion of Xanthomonas virulence factors. FEMS Microbiol Rev.

[74] Jang IA, Kim J, Park W (2016). Endogenous hydrogen peroxide increases biofilm formation by inducing exopolysaccharide production in Acinetobacter oleivorans DR1. Sci Rep.

[75] Oh E, Jeon B (2014). Role of alkyl hydroperoxide reductase (AhpC) in the biofilm formation of Campylobacter jejuni. PLoS One.

[76] Panmanee W, Hassett DJ (2009). Differential roles of OxyR-controlled antioxidant enzymes alkyl hydroperoxide reductase (AhpCF) and catalase (KatB) in the protection of Pseudomonas aeruginosa against hydrogen peroxide in biofilm vs. planktonic culture. FEMS Microbiol Lett.

[77] Wasim M, Bible AN, Xie Z, Alexandre G (2009). Alkyl hydroperoxide reductase has a role in oxidative stress resistance and in modulating changes in cell-surface properties in Azospirillum brasilense Sp245. Microbiology..

[78] Andrews S, Krueger F, Seconds-Pichon A, Biggins F, Wingett S. FastQC. A quality control tool for high throughput sequence data. Babraham Bioinformatics [Internet]. Vol. 1, Babraham Institute. 2015. p. 1. Available from: https://www.bioinformatics.babraham.ac.uk/projects/fastqc/, http://www.bioinformatics.bbsrc.ac.uk/projects/fastqc/

[79] Langmead B, Bowtie SS (2013). Nat Methods [Internet].

[80] Guarischi-Sousa R, Puigvert M, Coll NS, Siri MI, Pianzzola MJ, Valls M, et al. Complete genome sequence of the potato pathogen Ralstonia solanacearum UY031. Stand Genomic Sci 2016;11(1):1–8. 10.1186/s40793-016-0131-4 PMC4714475 26779304

[81] Anders S, Pyl PT, Huber W (2015). HTSeq-A Python framework to work with high-throughput sequencing data. Bioinformatics..

[82] Love MI, Huber W, Anders S (2014). Moderated estimation of fold change and dispersion for RNA-seq data with DESeq2. Genome Biol.

[83] R Team C. R Core Team (2017). R: a language and environment for statistical computing. R Found Stat Comput Vienna, Austria URL http//www R-project org/, page R Found Stat Comput 2017;

[84] Conway JR, Lex A, Gehlenborg N (2017). UpSetR: an R package for the visualization of intersecting sets and their properties. Bioinformatics..

[85] Kumar L, Futschik ME. Mfuzz: A software package for soft clustering of microarray data. Bioinformation. 2007. 10.6026/97320630002005 PMC2139991 18084642

[86] Götz S, García-Gómez JM, Terol J, Williams TD, Nagaraj SH, Nueda MJ (2008). High-throughput functional annotation and data mining with the Blast2GO suite. Nucleic Acids Res.

[87] Yu G, Wang LG, Han Y, He QY (2012). ClusterProfiler: an R package for comparing biological themes among gene clusters. Omi A J Integr Biol.

