# Supplementary figures and images for: Dynamic expression of Ralstonia solanacearum virulence factors and metabolism-controlling genes during plant infection
Source: BMC Genomics. 2021 Mar 9;22:170. doi: 10.1186/s12864-021-07457-w (PMC7941725; doi:10.1186/s12864-021-07457-w)

**A**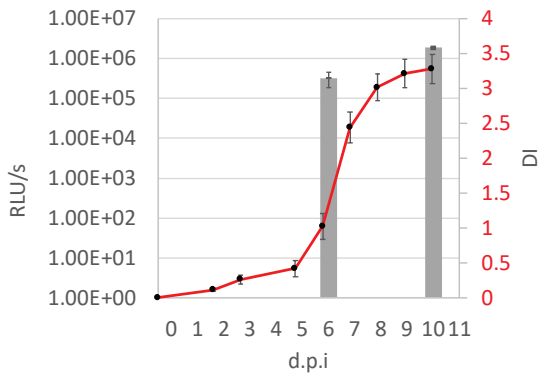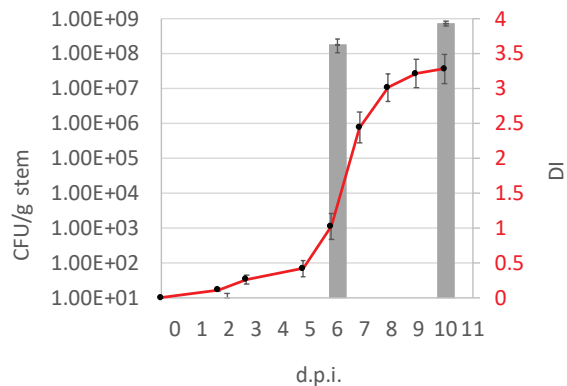**B**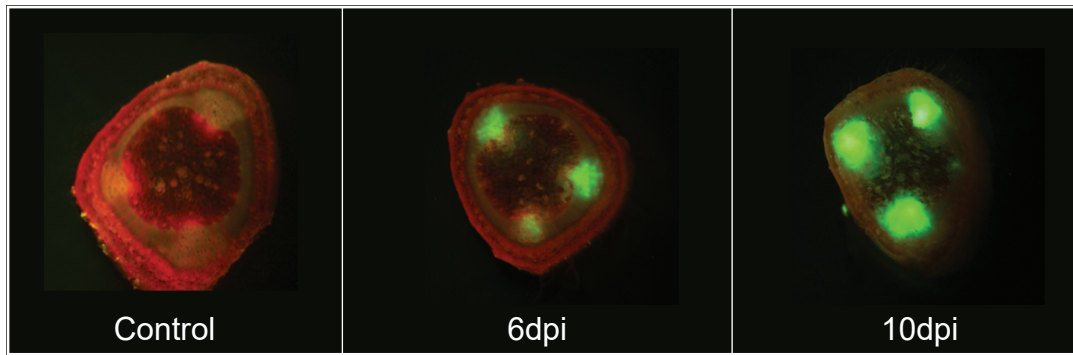

Supplement: Supplementary file 1 — Additional file 1: R. solanacearum reporter strains and bacterial growth show equivalent infection rates. (A) Luminescence levels or bacterial growth (bar plot) and symptom development (line plot) in potato plants were monitored over time to detect the precise time points at which similar bacterial yields but different symptoms could be detected. The disease index scale (DI) ranges from 0 to 4 being 0 symptomless plants and 4 plants completely wilted. Luminescence measurement were conducted on stem sections of infected plants. (B) GFP-labelled bacteria were monitored at the sampled time points in potato plants. RLU = Relative light units. [file 12864_2021_7457_MOESM1_ESM.pdf]

**A**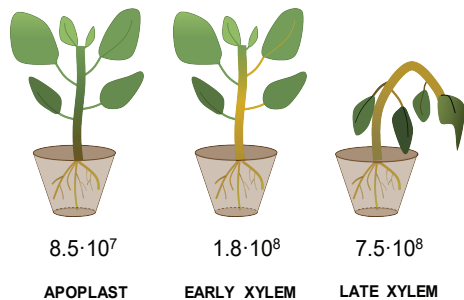**Reference**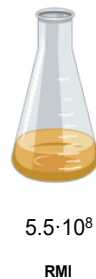**B**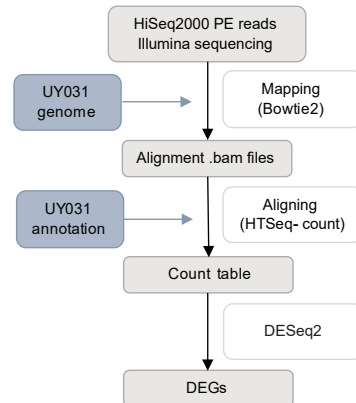**C**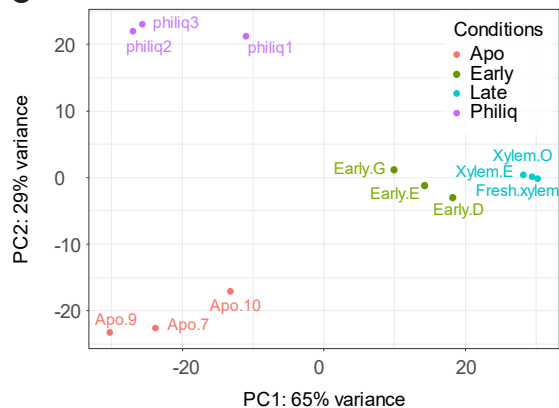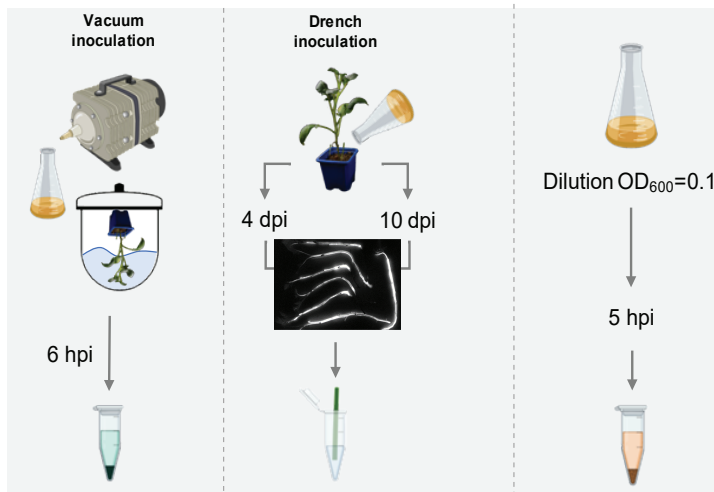

Supplement: Supplementary file 2 — Additional file 2: RNAseq experimental set-up and bioinformatic pipeline. (A) Experimental set-up for the three in planta conditions, corresponding to an early (leaf apoplast), mid (xylem from asymptomatic plants) and late stages (xylem from dead plants) of the disease. As reference condition, bacteria grown in rich liquid media were used. The average of bacterial yields recovered in each condition are indicated as CFU/ml. The grey background section of the figure contains the representation of how bacteria was enriched in each condition (see M&Ms). (B) Transcriptomic analysis pipeline. (C) Two-dimensional Principal Component Analysis representation of the expression data of the conditions’ biological replicates used in the study. [file 12864_2021_7457_MOESM2_ESM.pdf]

# Transcripts per million

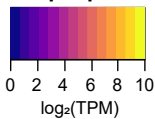

Rich B medium

Apoplast

Early xylem

Late xylem

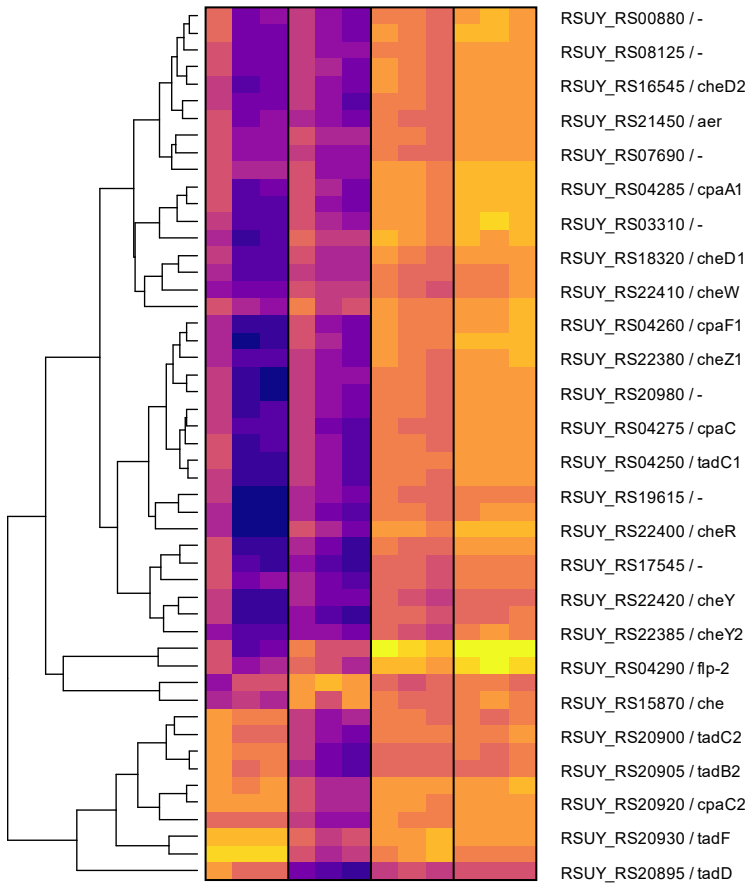

Supplement: Supplementary file 10 — Additional file 10: hemosensors and signal transduction gene expression profile. Heatmap showing the normalised transcripts per million (TPM) of the genes involved in chemosensing and signal transduction in the reference and in the in planta conditions. [file 12864_2021_7457_MOESM10_ESM.pdf]

Transcripts per million

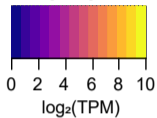

Rich B medium  
Apoplast  
Early xylem  
Late xylem

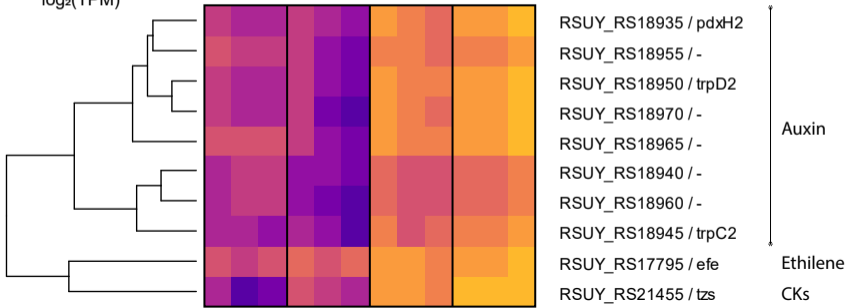

Supplement: Supplementary file 11 — Additional file 11: Phytohormones biosynthesis gene expression profile. Heatmap showing the normalised transcripts per million (TPM) of the genes involved in phytohormones biosynthesis in the reference and in the in planta conditions. [file 12864_2021_7457_MOESM11_ESM.pdf]
