# Supplementary material for: Dynamic expression of Ralstonia solanacearum virulence factors and metabolism-controlling genes during plant infection
Source: BMC Genomics. 2021 Mar 9;22:170. doi: 10.1186/s12864-021-07457-w (PMC7941725; doi:10.1186/s12864-021-07457-w)

A

Induced - UP						Repressed - DOWN							
Apoplast		Early Xylem		Late Xylem		Apoplast		Early Xylem		Late Xylem			Ref
**18%**	(40/226)	**9%**	(27/290)	**5%**	(20/378)	**0%**	(0)	**1%**	(3/241)	**5%**	(26/544)	**RNAseq UY031 - Potato root UP**	(36)
**0%**	(0)	**0%**	(1/290)	**0%**	(1/378)	**17%**	(32/192)	**9%**	(21/241)	**5%**	(26/544)	**RNAseq UY031 - Potato root DOWN**	(36)
**33%**	(74/226)	**34%**	(100/290)	**27%**	(102/378)	**6%**	(11/192)	**3%**	(8/241)	**2%**	(12/544)	**µarray UW551 - Tomato UP**	(14)
**1%**	(2/226)	**1%**	(4/290)	**1%**	(4/378)	**32%**	(61/192)	**36%**	(86/241)	**26%**	(142/544)	**µarray UW551 - Tomato DOWN**	(14)
**13%**	(30/226)	**20%**	(58/290)	**15%**	(56/378)	**5%**	(9/192)	**3%**	(8/241)	**2%**	(9/544)	**µarray GMI1000 - Tomato UP**	(14)
**1%**	(2/226)	**1%**	(2/290)	**1%**	(2/378)	**16%**	(31/226)	**21%**	(50/241)	**16%**	(86/544)	**µarray GMI1000 - Tomato DOWN**	(14)
**8%**	(17/226)	**9%**	(27/290)	**7%**	(27/378)	**6%**	(12/226)	**5%**	(11/241)	**3%**	(16/544)	**RNAseq GMI1000 - Tomato UP**	(42)
**5%**	(12/226)	**5%**	(14/290)	**4%**	(15/378)	**15%**	(29/226)	**8%**	(19/241)	**12%**	(63/544)	**RNAseq GMI1000 - Tomato DOWN**	(42)

B

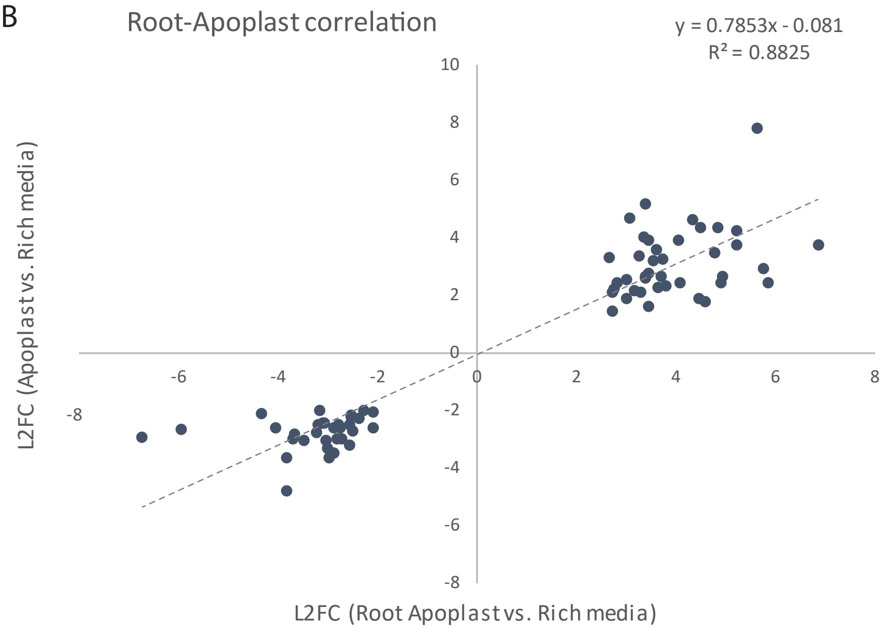

Supplement: Supplementary file 4 — Additional file 4: Overlap of DEGs in apoplast, early and late xylem compared to previous gene expression analysis. (A) Percentage of common DE genes in each in planta condition (versus rich medium) compared to previous in planta gene expression analyses (− Puigvert et al. 2017; −Jacobs et al. 2012; −Khokhani et al. 2017). Fractions represent the overlapping genes from the total of DEGs in each of our conditions compared to a given previous gene expression analysis. Colors were plotted using the Conditional Formatting tool in Microsoft Excel. (B) Expression correlation of the DE data of the common genes between our Apoplast data and the RNAseq data from the potato root (Puigvert et al. 20,107). [file 12864_2021_7457_MOESM4_ESM.pdf]
