# Supplementary material for: Dynamic expression of Ralstonia solanacearum virulence factors and metabolism-controlling genes during plant infection
Source: BMC Genomics. 2021 Mar 9;22:170. doi: 10.1186/s12864-021-07457-w (PMC7941725; doi:10.1186/s12864-021-07457-w)
Supplement: Supplementary file 5 — Additional file 5: Transcriptomic profile of R. solanacearum in in planta genetic programmes. Up-regulated (left) and down-regulated (right) genes shared and unique across the three in planta conditions. Each vertical bar plot represents the number of shared DE between the conditions indicated by the lines and dots in the schematic below. The horizontal bar plots on the right indicate the total DE genes per in planta condition compared to rich medium. For the intersection of Apoplast, Early and Late (in planta environment), Early and Late (Xylem environment), Apoplast and Late xylem alone, the list of genes was extracted and surveyed for enriched GO terms. Dot plots of the enriched GO terms for the up- (left) and down-regulated (right) genes in each environment is shown below. DE genes were identified with DEseq2 (p-adj > 0.01, log2 FC ± 1.5) and plotted using the R package UpsetR. [file 12864_2021_7457_MOESM5_ESM.pdf]

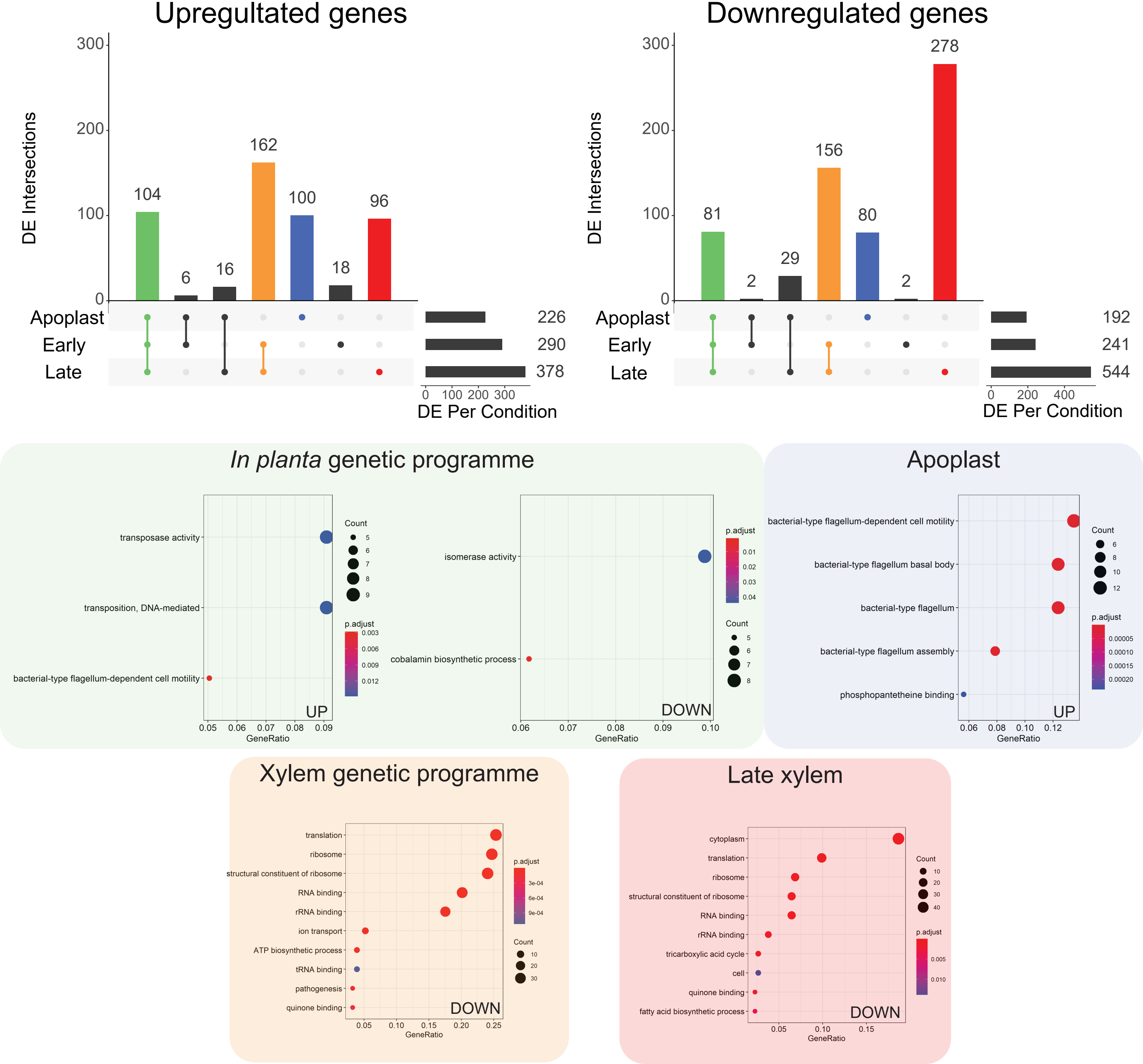

Upregultated genes
Downregulated genes
DE Intersections
104
6
16
162
100
18
96
81
2
29
156
80
2
278
Apoplast
Early
Late
226
290
378
192
241
544
DE Per Condition
In planta genetic programme
transposase activity
transposition, DNA-mediated
bacterial-type flagellum-dependent cell motility
UP
isomerase activity
cobalamin biosynthetic process
DOWN
Count
p.adjust
GeneRatio
Apoplast
bacterial-type flagellum-dependent cell motility
bacterial-type flagellum basal body
bacterial-type flagellum
bacterial-type flagellum assembly
phosphopantetheine binding
UP
Xylem genetic programme
translation
ribosome
structural constituent of ribosome
RNA binding
rRNA binding
ion transport
ATP biosynthetic process
tRNA binding
pathogenesis
quinone binding
DOWN
Late xylem
cytoplasm
translation
ribosome
structural constituent of ribosome
RNA binding
rRNA binding
tricarboxylic acid cycle
cell
quinone binding
fatty acid biosynthetic process
DOWN
