# Supplementary material for: Dynamic expression of Ralstonia solanacearum virulence factors and metabolism-controlling genes during plant infection
Source: BMC Genomics. 2021 Mar 9;22:170. doi: 10.1186/s12864-021-07457-w (PMC7941725; doi:10.1186/s12864-021-07457-w)
Supplement: Supplementary file 8 — Additional file 8: Gene expression dynamics of R. solanacearum throughout infection. Six clusters were obtained through Mfuzz clustering of log2- fold-change data of the apoplast, early and late xylem conditions normalised to the reference rich liquid media. Clusters include the genes (number indicated above each graph) with a membership higher than 70% and consistently associated to the same cluster on at least 30 out of 40 iterations. The list of genes associated to each cluster was extracted and surveyed for enriched GO terms. Dot plots of the enriched GO terms in each cluster is shown next to the cluster. [file 12864_2021_7457_MOESM8_ESM.pdf]

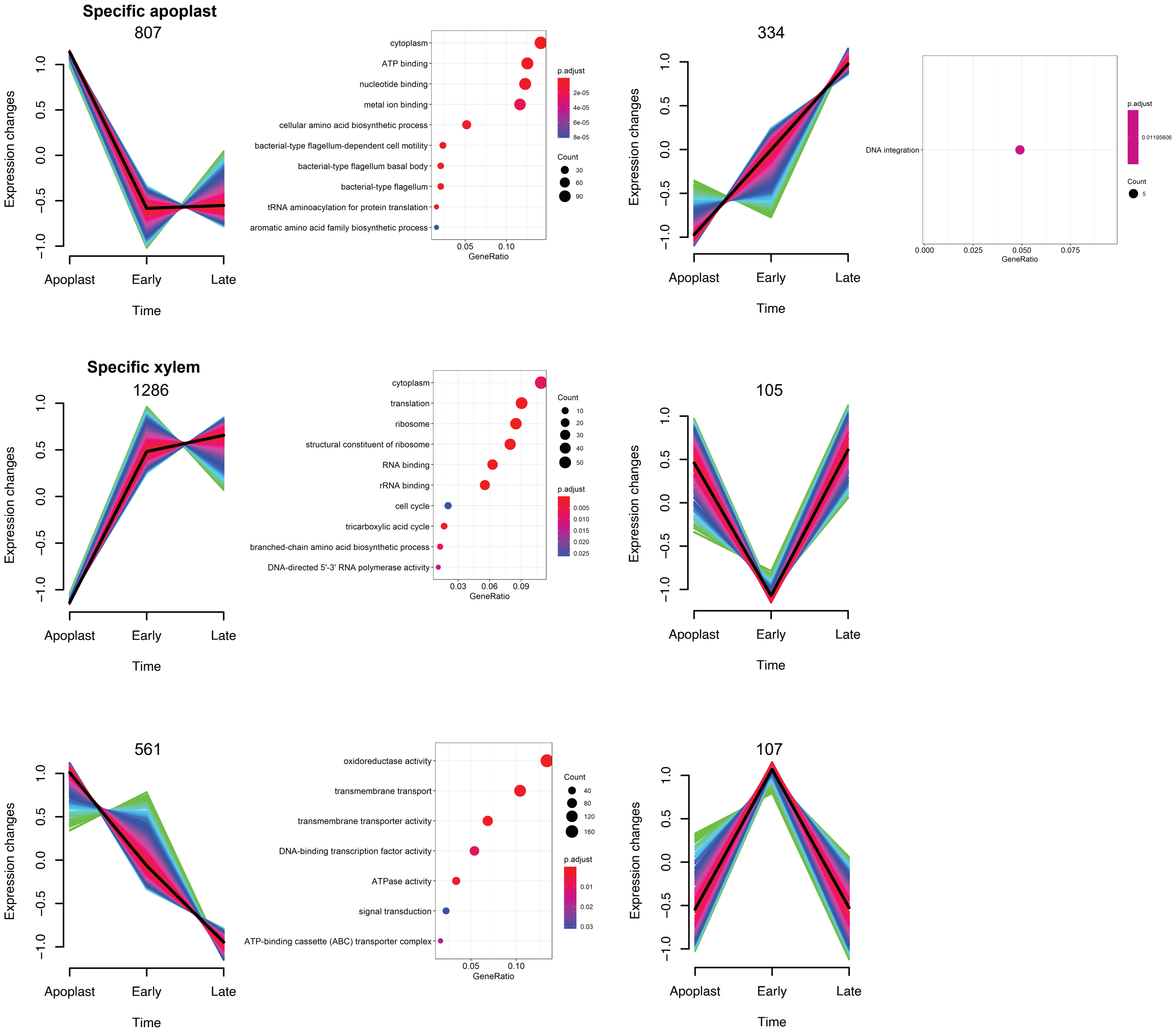
Specific apoplast
807
Expression changes
Apoplast
Early
Late
Time
cytoplasm
ATP binding
nucleotide binding
metal ion binding
cellular amino acid biosynthetic process
bacterial-type flagellum-dependent cell motility
bacterial-type flagellum basal body
bacterial-type flagellum
tRNA aminoacylation for protein translation
aromatic amino acid family biosynthetic process
GeneRatio
p.adjust
Count
334
DNA integration
Specific xylem
1286
translation
ribosome
structural constituent of ribosome
RNA binding
rRNA binding
cell cycle
tricarboxylic acid cycle
branched-chain amino acid biosynthetic process
DNA-directed 5'-3' RNA polymerase activity
105
561
oxidoreductase activity
transmembrane transport
transmembrane transporter activity
DNA-binding transcription factor activity
ATPase activity
signal transduction
ATP-binding cassette (ABC) transporter complex
107
