# Supplementary material for: Dynamic expression of Ralstonia solanacearum virulence factors and metabolism-controlling genes during plant infection
Source: BMC Genomics. 2021 Mar 9;22:170. doi: 10.1186/s12864-021-07457-w (PMC7941725; doi:10.1186/s12864-021-07457-w)
Supplement: Supplementary file 9 — Additional file 9: T3SS regulatory cascade and apparatus gene expression profile. Heatmap showing the normalised transcripts per million (TPM) of the genes involved in the T3SS regulatory cascade and the T3SS apparatus in the reference and in the in planta conditions. [file 12864_2021_7457_MOESM9_ESM.pdf]

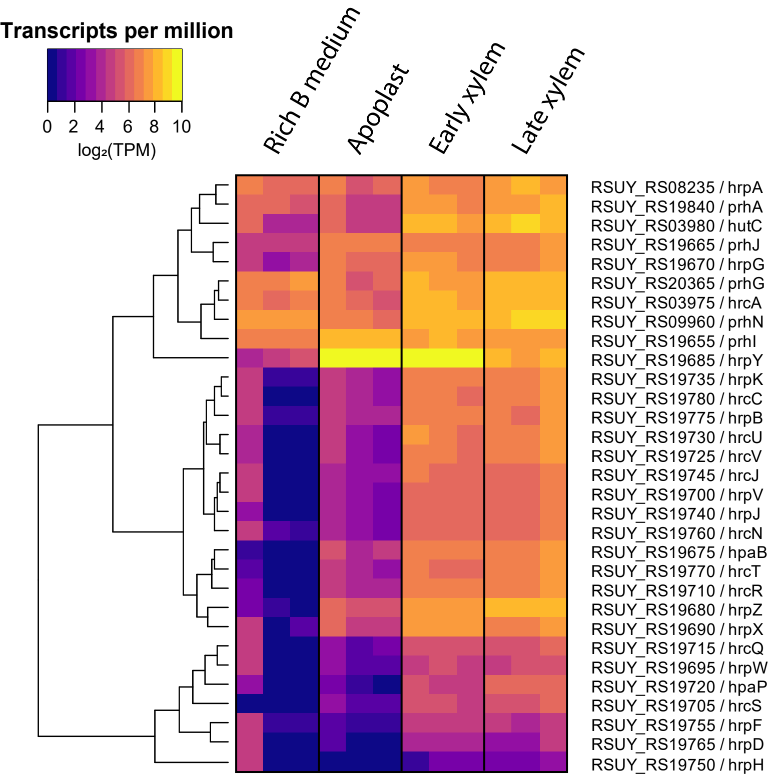

Transcripts per million
0
2
4
6
8
10
log2(TPM)
Rich B medium
Apoplast
Early xylem
Late xylem
RSUY_RS08235 / hrpA
RSUY_RS19840 / prhA
RSUY_RS03980 / hutC
RSUY_RS19665 / prhJ
RSUY_RS19670 / hrpG
RSUY_RS20365 / prhG
RSUY_RS03975 / hrcA
RSUY_RS09960 / prhN
RSUY_RS19655 / prhI
RSUY_RS19685 / hrpY
RSUY_RS19735 / hrpK
RSUY_RS19780 / hrcC
RSUY_RS19775 / hrpB
RSUY_RS19730 / hrcU
RSUY_RS19725 / hrcV
RSUY_RS19745 / hrcJ
RSUY_RS19700 / hrpV
RSUY_RS19740 / hrpJ
RSUY_RS19760 / hrcN
RSUY_RS19675 / hpaB
RSUY_RS19770 / hrcT
RSUY_RS19710 / hrcR
RSUY_RS19680 / hrpZ
RSUY_RS19690 / hrpX
RSUY_RS19715 / hrcQ
RSUY_RS19695 / hrpW
RSUY_RS19720 / hpaP
RSUY_RS19705 / hrcS
RSUY_RS19755 / hrpF
RSUY_RS19765 / hrpD
RSUY_RS19750 / hrpH
