# Supplementary material for: Dynamic expression of Ralstonia solanacearum virulence factors and metabolism-controlling genes during plant infection
Source: BMC Genomics. 2021 Mar 9;22:170. doi: 10.1186/s12864-021-07457-w (PMC7941725; doi:10.1186/s12864-021-07457-w)
Supplement: Supplementary file 12 — Additional file 12: ROS scavenging enzymes gene expression profile. Heatmap showing the normalised transcripts per million (TPM) of the genes coding for ROS scavenging enzymes in the reference condition and in planta apoplast, early and late condition. [file 12864_2021_7457_MOESM12_ESM.pdf]

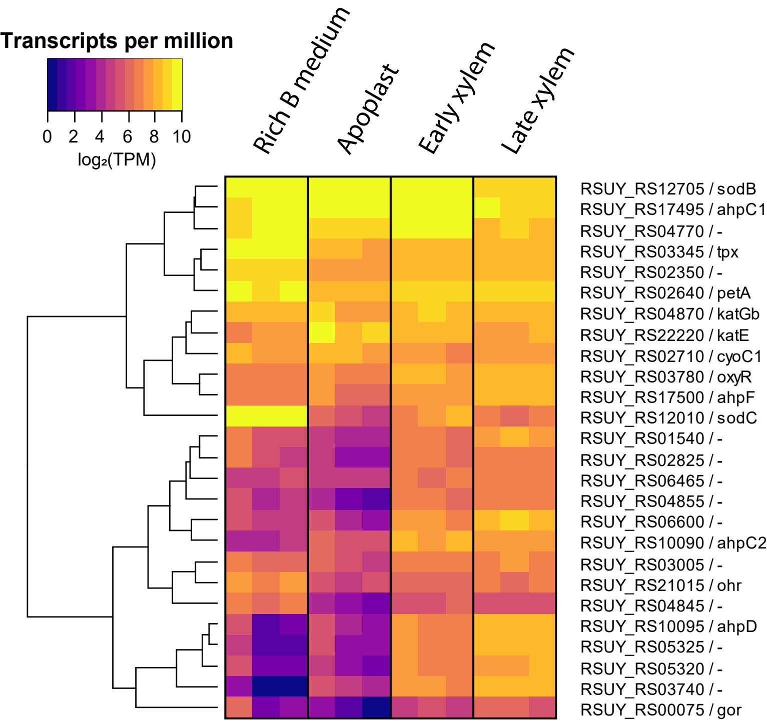
Transcripts per million
0
2
4
6
8
10
log₂(TPM)
Rich B medium
Apoplast
Early xylem
Late xylem
RSUY_RS12705 / sodB
RSUY_RS17495 / ahpC1
RSUY_RS04770 / -
RSUY_RS03345 / tpx
RSUY_RS02350 / -
RSUY_RS02640 / petA
RSUY_RS04870 / katGb
RSUY_RS22220 / katE
RSUY_RS02710 / cyoC1
RSUY_RS03780 / oxyR
RSUY_RS17500 / ahpF
RSUY_RS12010 / sodC
RSUY_RS01540 / -
RSUY_RS02825 / -
RSUY_RS06465 / -
RSUY_RS04855 / -
RSUY_RS06600 / -
RSUY_RS10090 / ahpC2
RSUY_RS03005 / -
RSUY_RS21015 / ohr
RSUY_RS04845 / -
RSUY_RS10095 / ahpD
RSUY_RS05325 / -
RSUY_RS05320 / -
RSUY_RS03740 / -
RSUY_RS00075 / gor
